# Supplementary material for: Structural insights into substrate recognition by the SOCS2 E3 ubiquitin ligase
Source: Nat Commun. 2019 Jun 10;10:2534. doi: 10.1038/s41467-019-10190-4 (PMC6557900; doi:10.1038/s41467-019-10190-4)
Supplement: Supplementary file 1 — Supplementary Information [file 41467_2019_10190_MOESM1_ESM.pdf]

Supplementary Information

Structural insights into substrate recognition by the SOCS2 E3 ubiquitin ligase

Wei-Wei Kung, Sarath Ramachandran, Nikolai Makukhin et al.

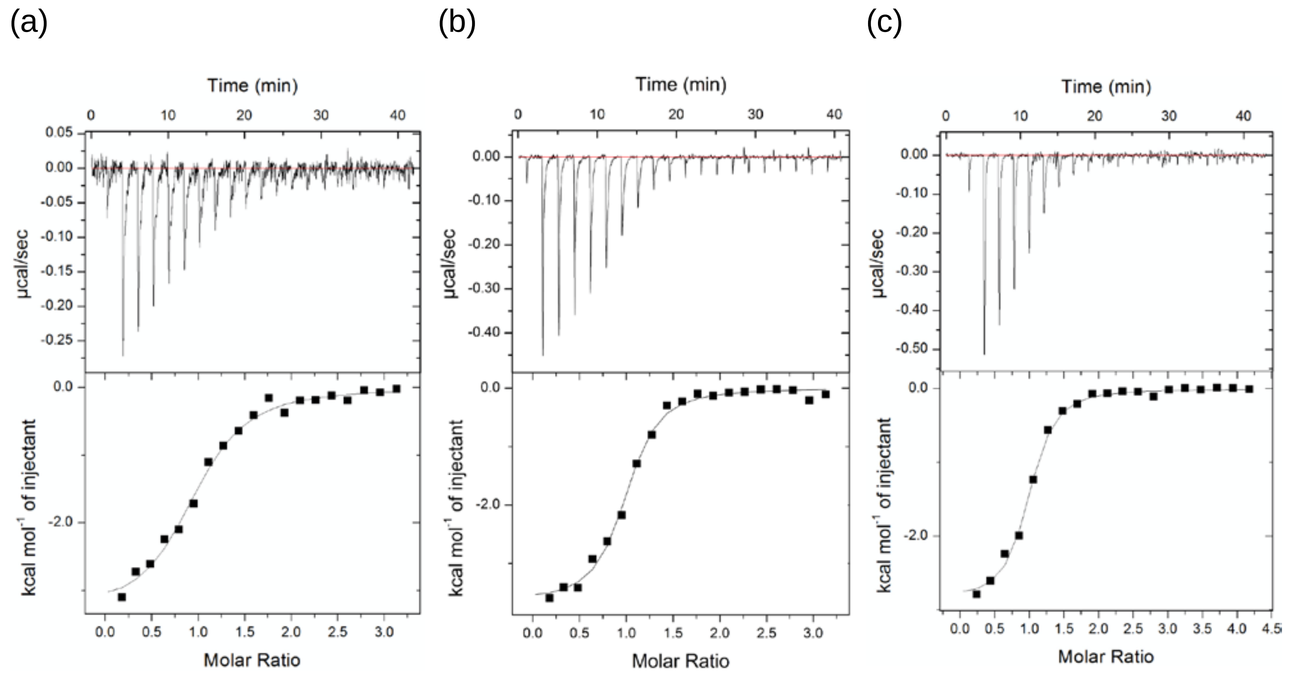

(d)

SBC v/s	No. of experiments	K_D (µM)	ΔG (kcal × mol⁻¹)	ΔH (kcal × mol⁻¹)	TΔS (kcal × mol⁻¹)	Stoichiometry (N)
**GHR_pY595***	**4**	**1.11 ± 0.15**	**-8.12 ± 0.08**	**-3.48 ± 0.07**	**4.63 ± 0.08**	**1.03 ± 0.03**
EpoR_pY426$	7	6.92 ± 1.27	-7.04 ± 0.11	-3.14 ± 0.18	3.84 ± 0.21	1.11 ± 0.04
**GHR_pY487#**	**3**	**2.30 ± 0.37**	**-7.69 ± 0.09**	**-2.82± 0.06**	**4.84± 0.11**	**0.93 ± 0.03**

*GHR_pY595, PVPDpYTSIHIV-amide
$EpoR_pY426, ASFEpYTILDPS-amide
#GHR_pY487, NIDFpYAQVSDI -amide

Supplementary Figure 1. Biophysical characterisation of the interactions between SBC and phosphorylated substrate peptides

ITC measurement of (a) the EpoR_pY426 peptide (b) GHR_pY595 peptide and (c) GHR_pY487 peptide binding to the SOCS2-EloB-EloC ternary (SBC) complex at 298K. (d) ITC binding data for phosphorylated substrate peptides. Values reported are the means ± s.e.m. from independent experiments.

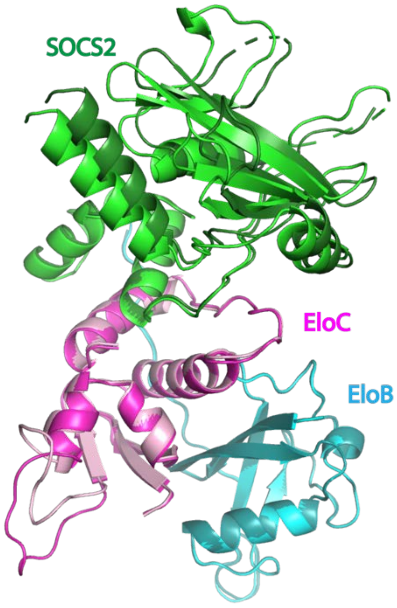

Supplementary Figure 2. The hinge motion of SOCS2

Superposition of the two protomers from SBC-GHR structure via EloB (cyan and blue) backbone atom alignment. A hinge motion of SOCS2 (green and dark green) is observed.

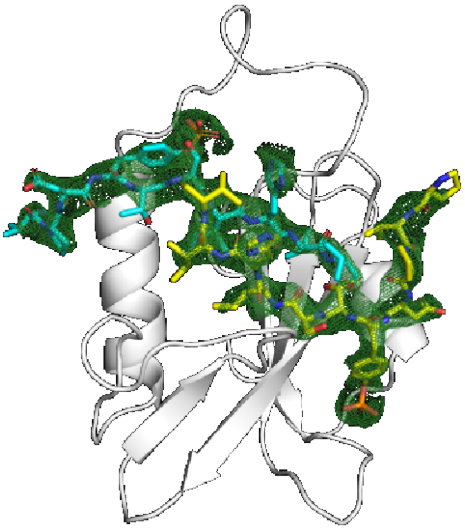

Supplementary Figure 3. Structural detail of the SBC-GHR$_2$ co-crystal

The Fo-Fc ligand omit map of the GHR peptides (green mesh) contoured at 2.5 σ level to highlight densities for the GHR_pY595 peptides (yellow and cyan stick).

(a)

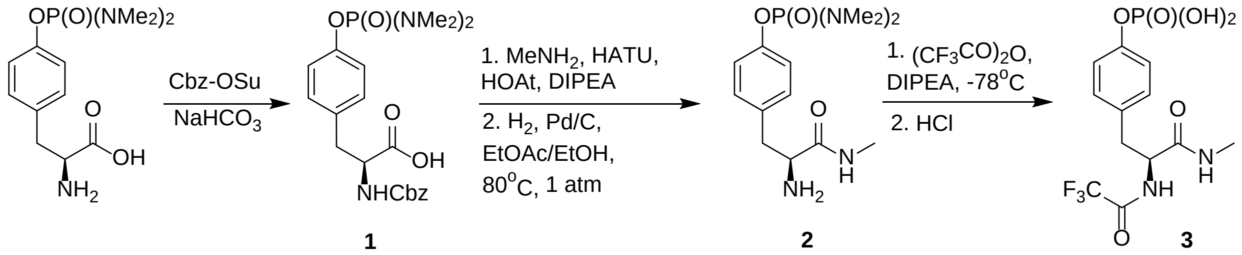

(b)

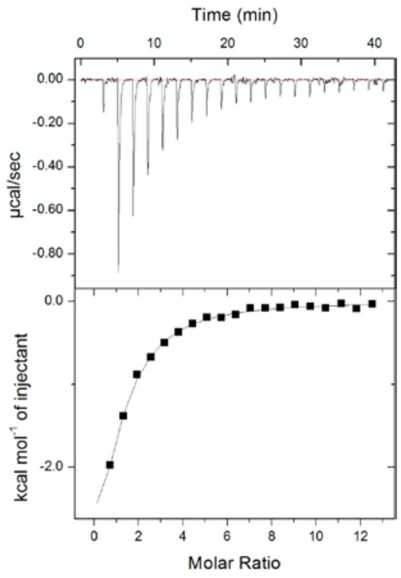

SBC v/s	No. of experiments	K_D (µM)	Δ*G* (kcal × mol⁻¹)	Δ*H* (kcal × mol⁻¹)	TΔ*S* (kcal × mol⁻¹)	Stoichiometry (N)
**Spy**	4	50 ± 4.44	-5.86 ± 0.05	-5.34 ± 0.26	0.52 ± 0.27	1.00 ± 0.09

(c)

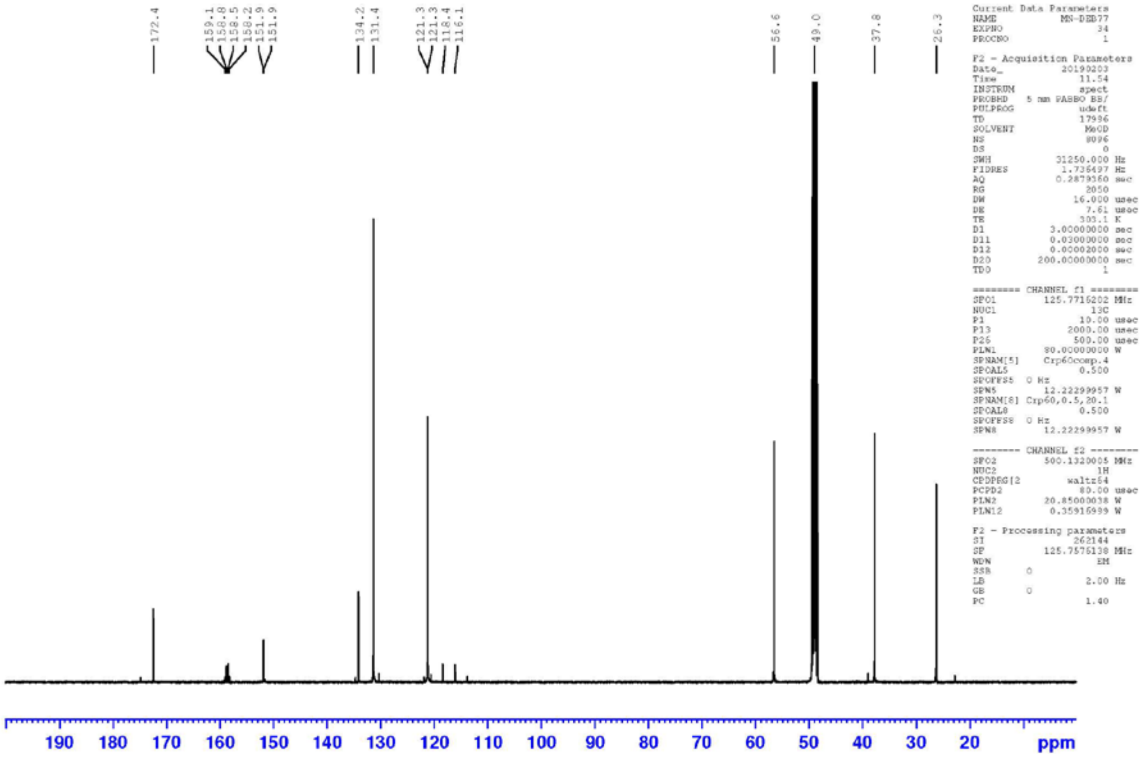

(d)

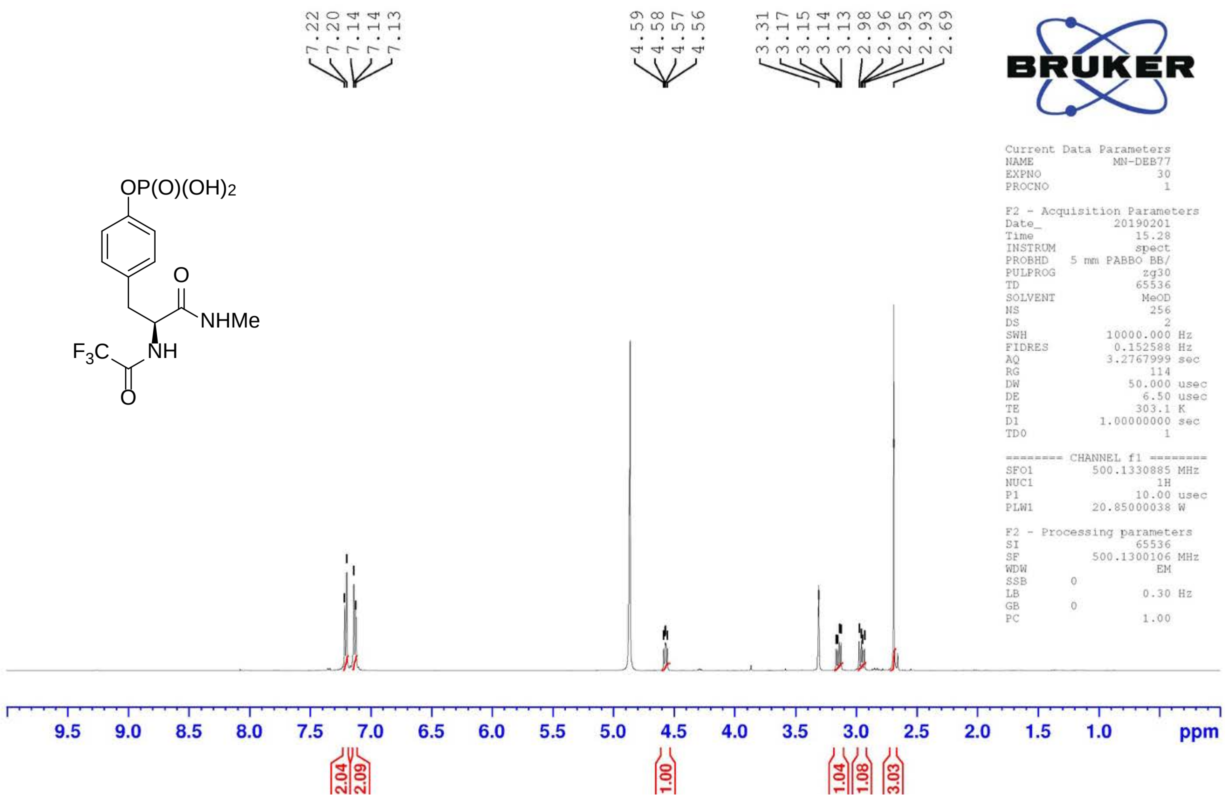

(e)

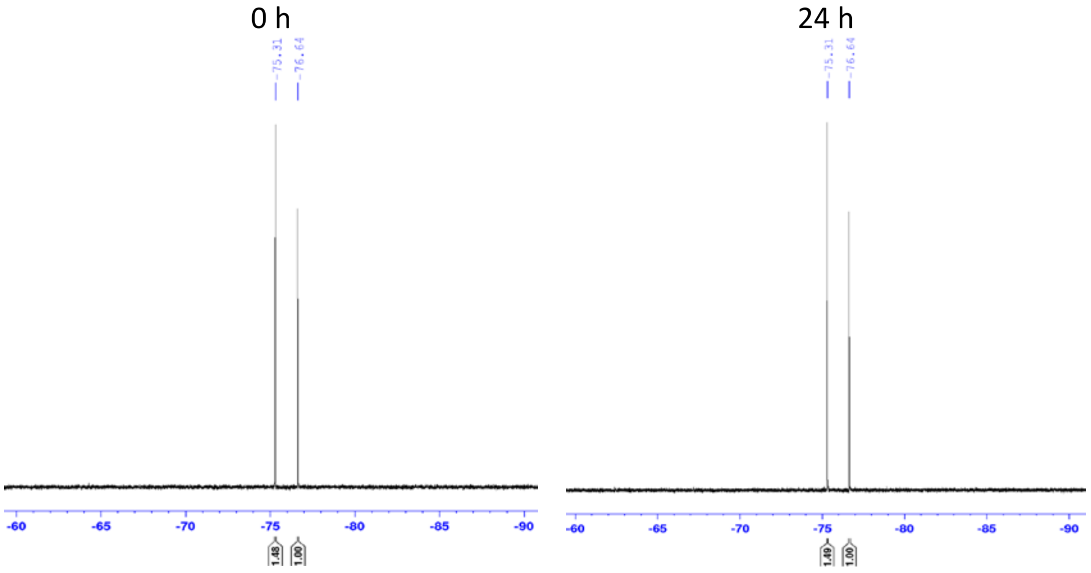

Supplementary Figure 4. Characterization of spy molecule 3
(a) Synthesis of spy molecule **3** (b) Binding affinity of the phosphate 3 to SBC determined by ITC. The ITC measurement was carried out at 298K. Values reported are the means ± s.e.m. from four independent experiments (c) ^{13}C NMR (500 MHz, CD_3OD) of phosphate **3** (d) ^{1}H NMR (500 MHz, CD_3OD) of phosphate **3** (e) Phosphate **3** stability test. The two peaks are phosphate **3** and an internal reference trifluoroethanol in NMR buffer (20 mM HEPES, pH8, 50 mM NaCl and 1 mM DTT) measured at 0 and 24 h at room temperature.

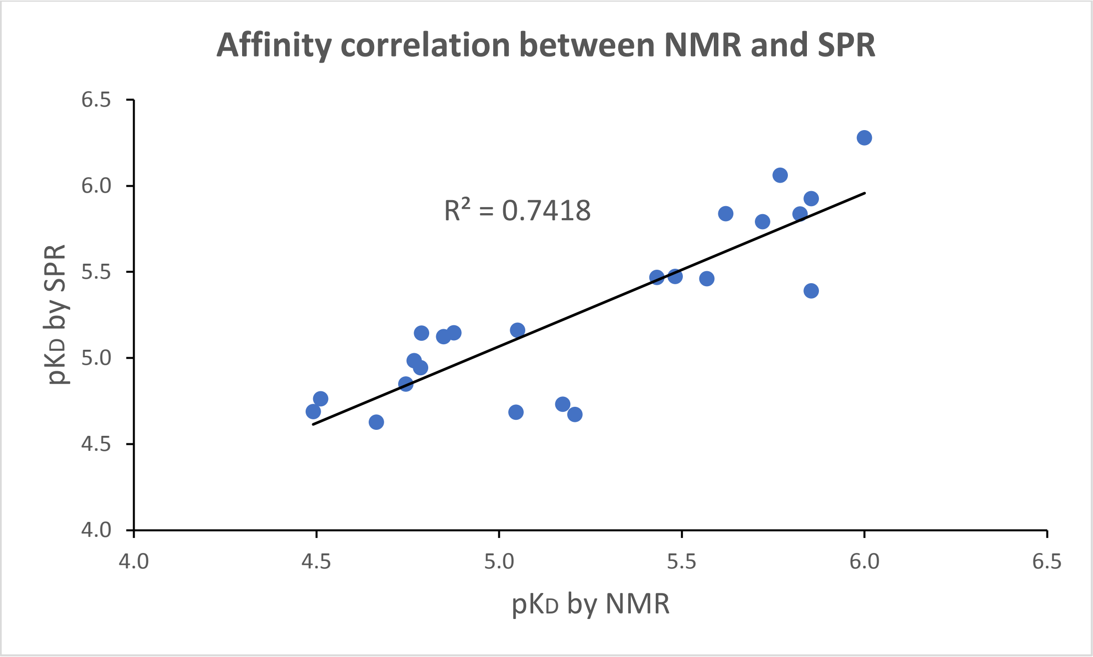

Supplementary Figure 5. Correlation of binding affinity of the alanine peptide library measured by ^{19}F-NMR and SPR technique

Spectra from 10 to –3 ppm

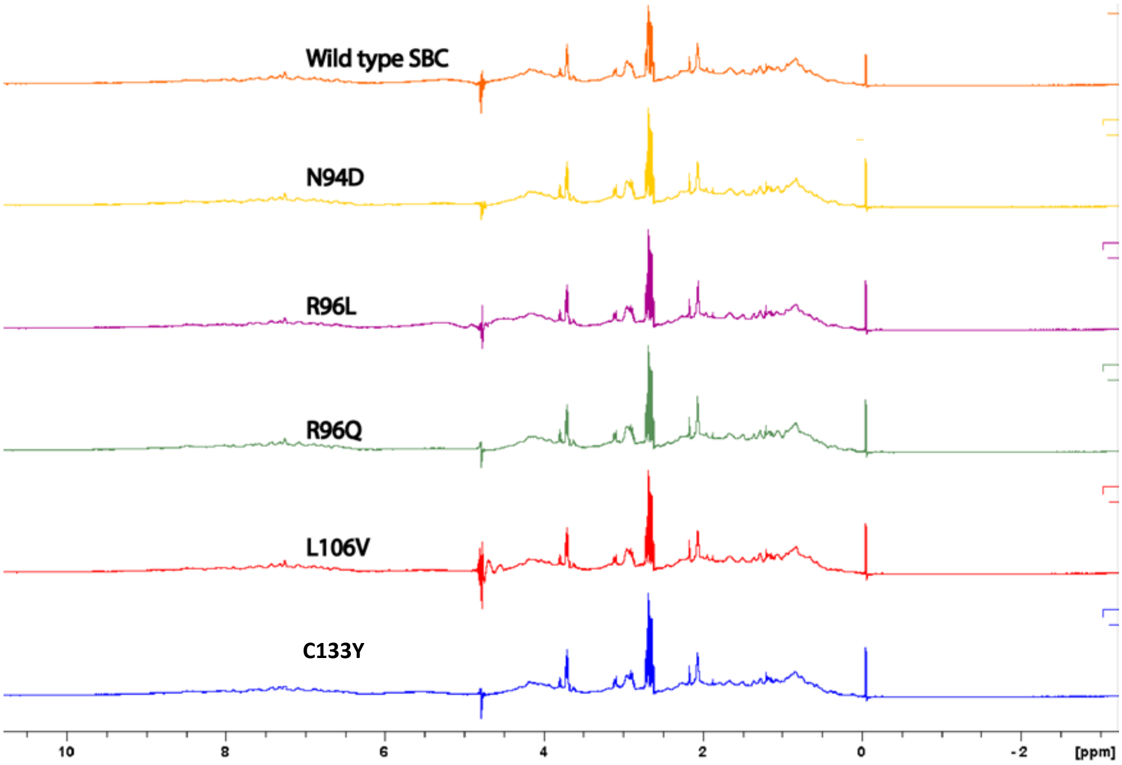

Spectra zoom in from 10 to 6 ppm

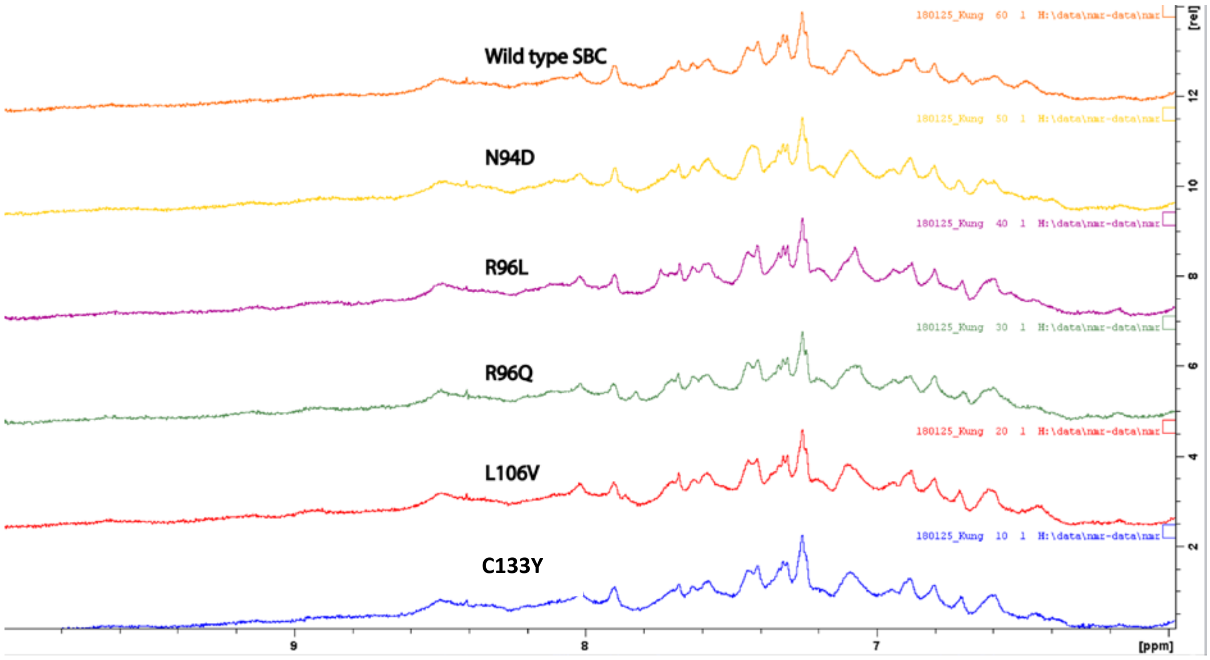

Spectra zoom in from 1.5 to –0.5 ppm

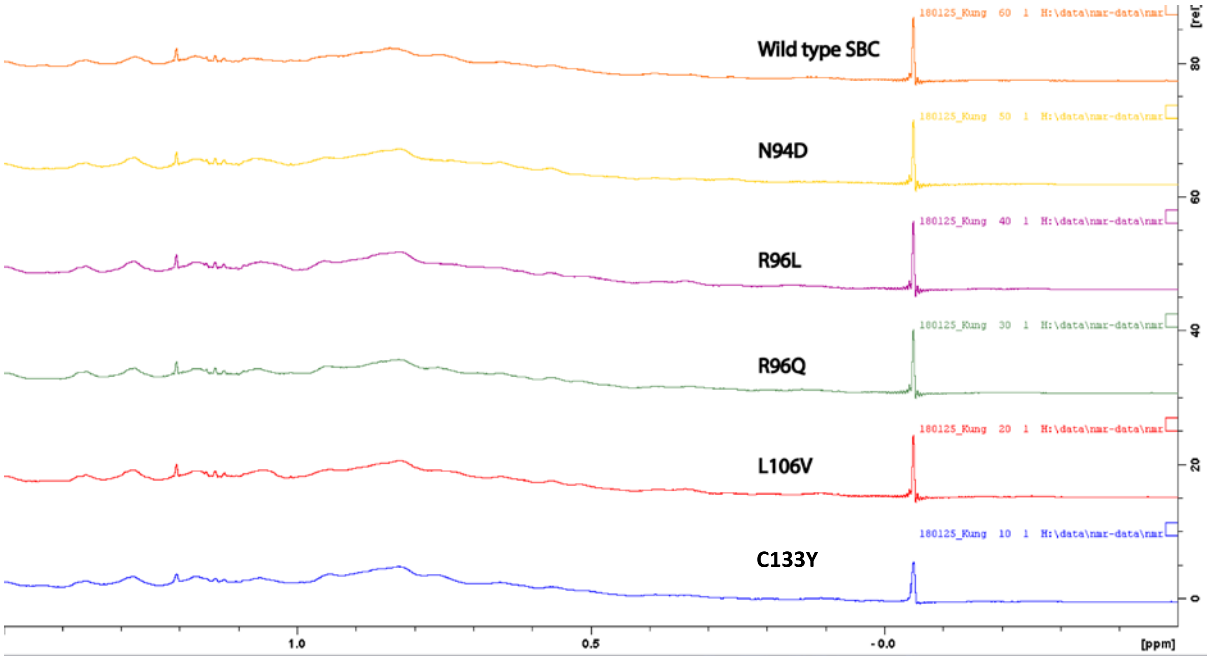

Supplementary Figure 6. The protein folding of SNPs containing SOCS2 confirmed by ^{1}H 1D NMR

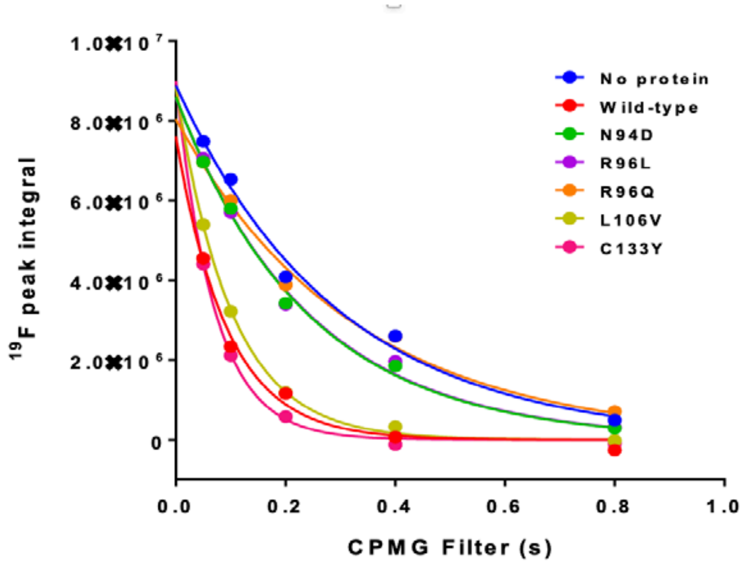

One phase decay	No protein	Wild-type	N94D	R96L	R96Q	L106V	C133Y
Best-fit values							
Y0	8885000	7611000	8593000	8572000	8040000	8857000	8997000
R_2	3.402	10.75	4.179	4.136	3.114	9.999	14.33
T_2	0.294	0.09303	0.2393	0.2418	0.3212	0.1	0.06981
Std. Error							
Y0	418918	920328	336002	404716	369681	305191	545388
K	0.3361	1.631	0.3177	0.3811	0.3094	0.45	0.92
R square	0.9899	0.9853	0.9946	0.9919	0.9889	0.9986	0.9975

Supplementary Figure 7. Measurement of the transverse relaxation rate (R_2) of the spy molecule in the absence and presence of protein. Data fitted by prism.

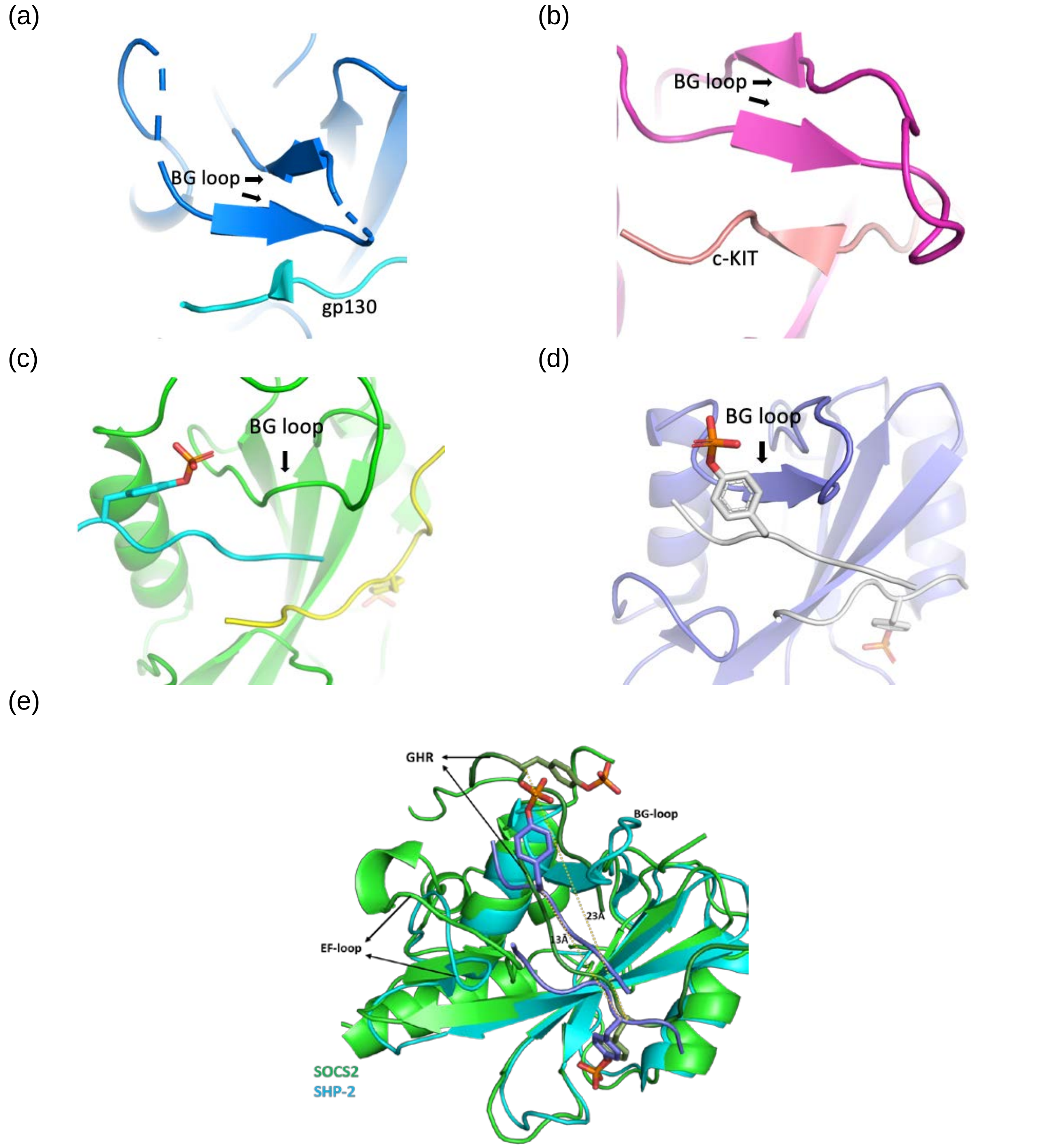

Supplementary Figure 8. The triple-stranded β-sheet interaction between substrate peptide and protein

(a) The SOCS3 (blue) in complex with gp130 (cyan) (PDB code: 2HMH) (B) The SOCS6 (magenta) in complex with c-Kit (pink) (PBD code: 2VIF) (C) The SOCS2 (green) in complex with GHR_pY595 peptides. Peptide A is shown in yellow and peptide B in cyan. (d) The SHP2 (purple) in complex with peptides (white). (e) overlap of SH2 domains from SOCS2-GHR (green) and SHP2-pY peptide (cyan) highlights the difference in positioning of non-canonical pY from GHR and SHP2-substrate peptide

AVPDpYTSIHIV (MW~1293.581)

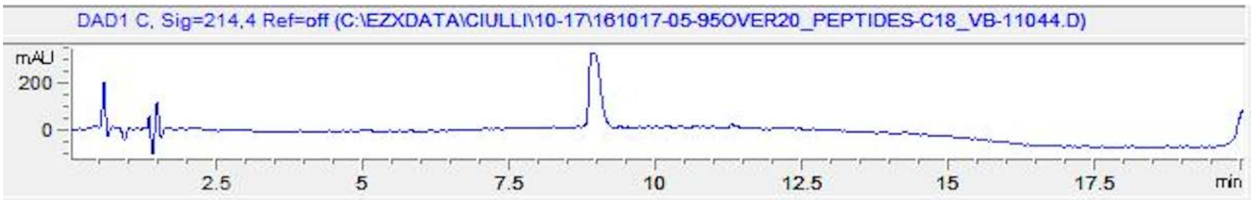

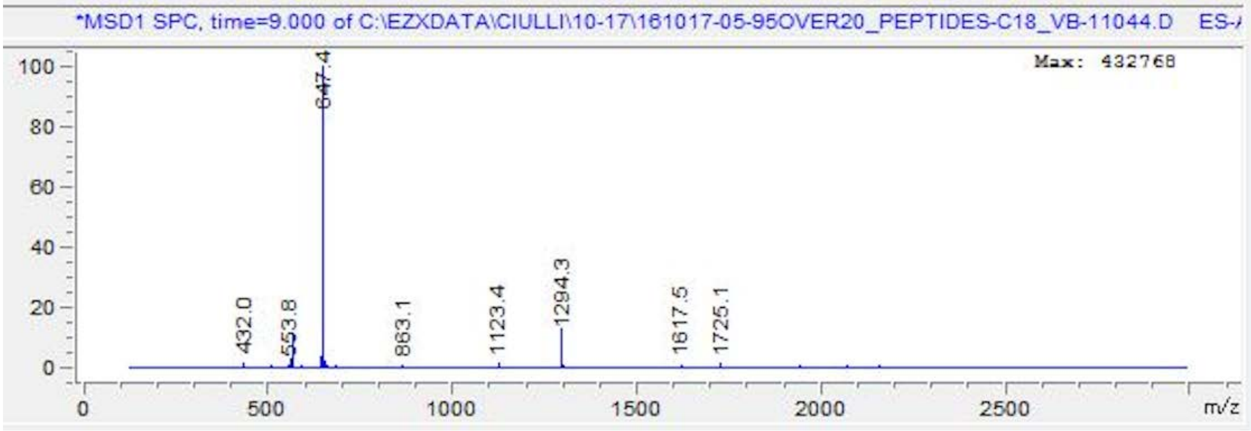

PAPDpYTSIHIV (MW~1291.565)

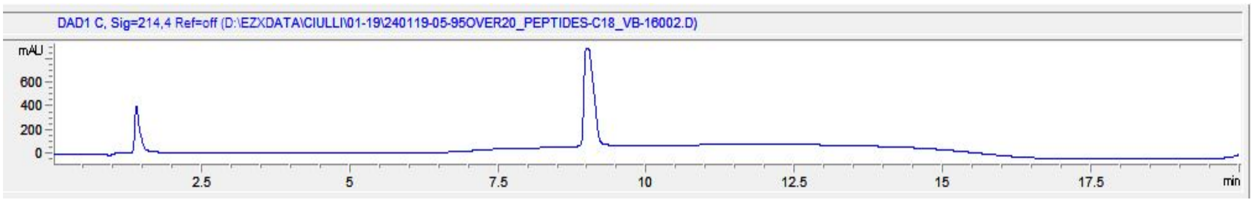

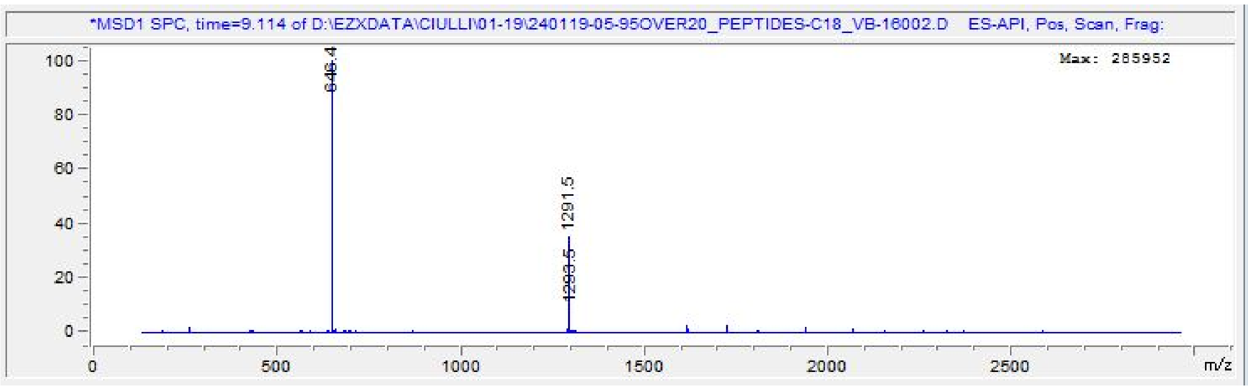

PVADpYTSIHIV (MW~1293.581)

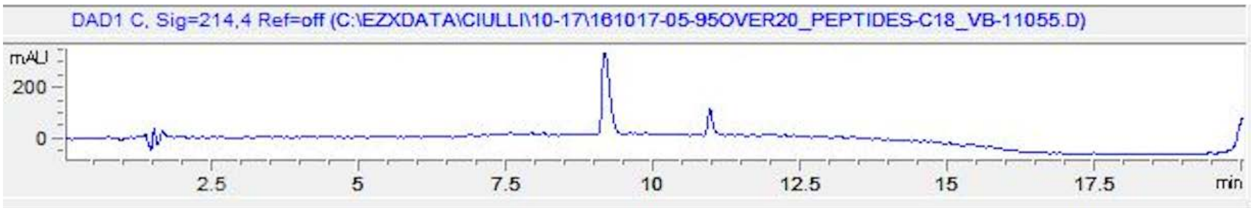

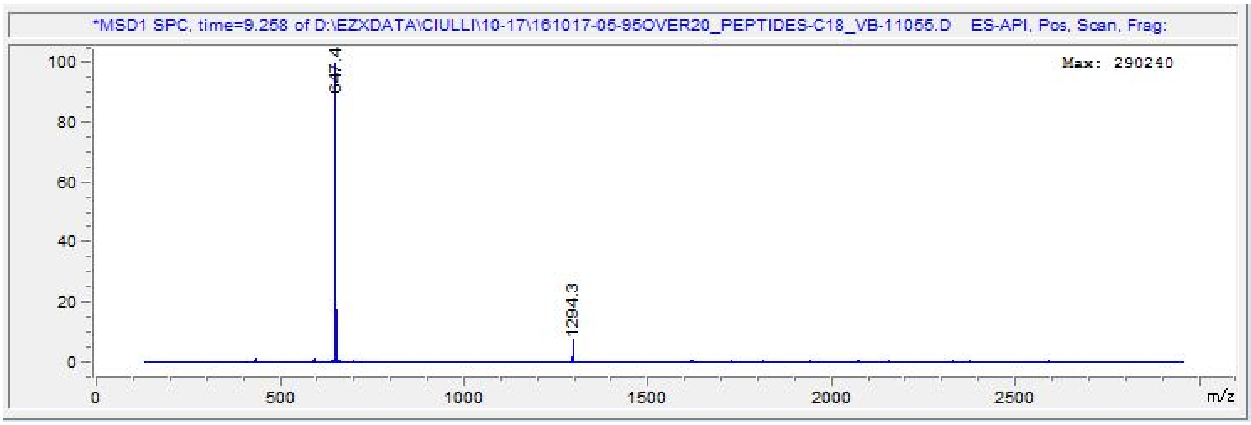

PVPApYTSIHIV (MW~1275.607)

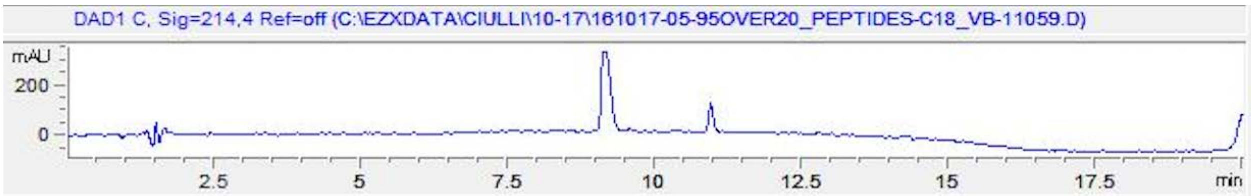

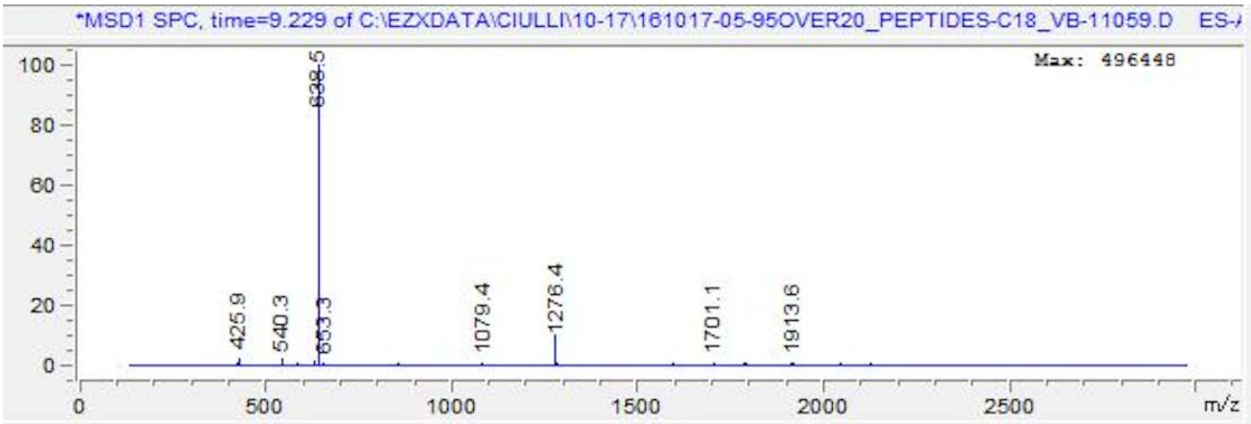

PVPDpYASIHIV (MW~1289.586)

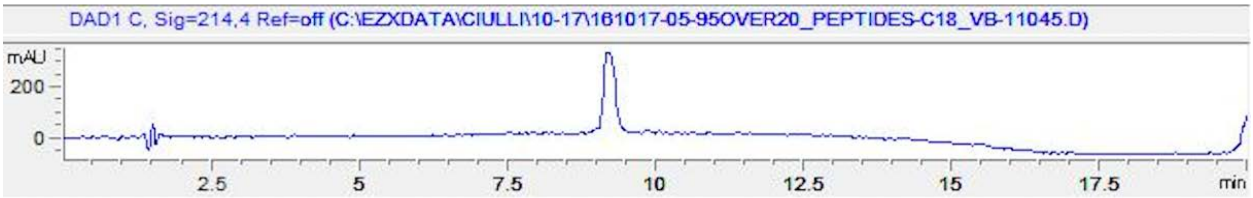

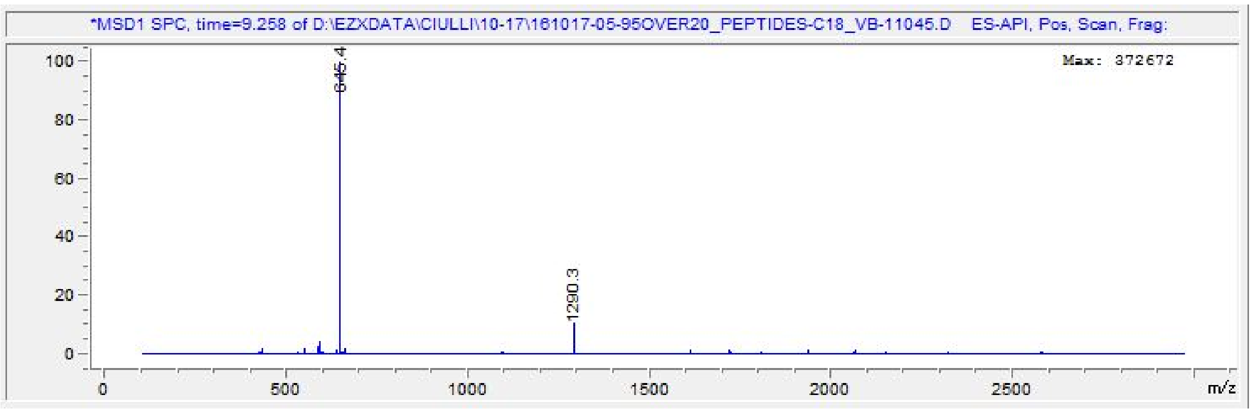

PVPDpYTAIHIV (MW~1303.602)

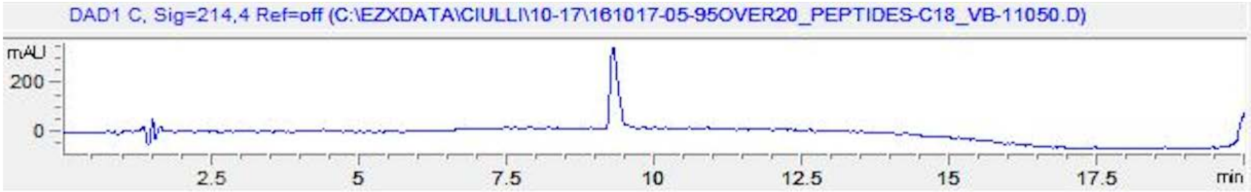

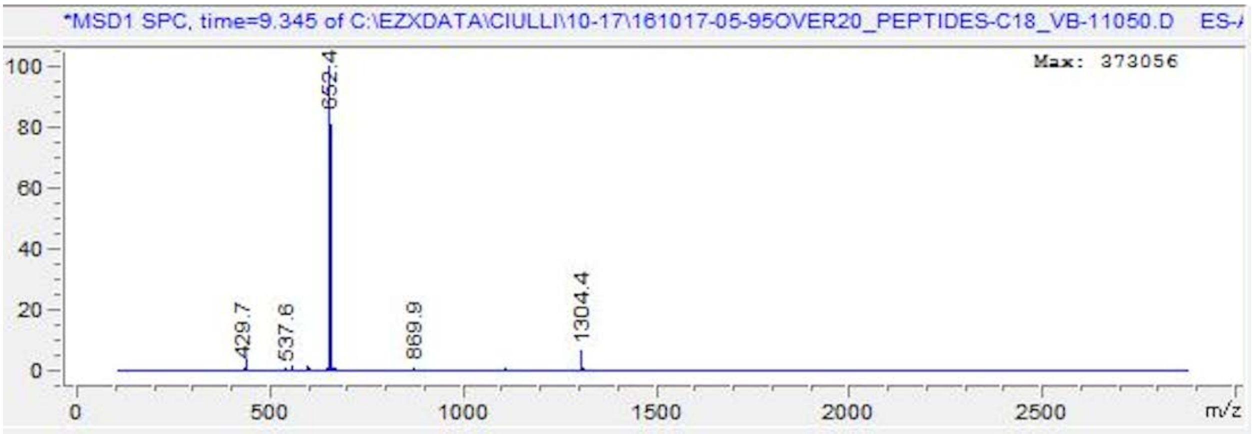

PVPDpYTSAHIV (MW~1277.550)

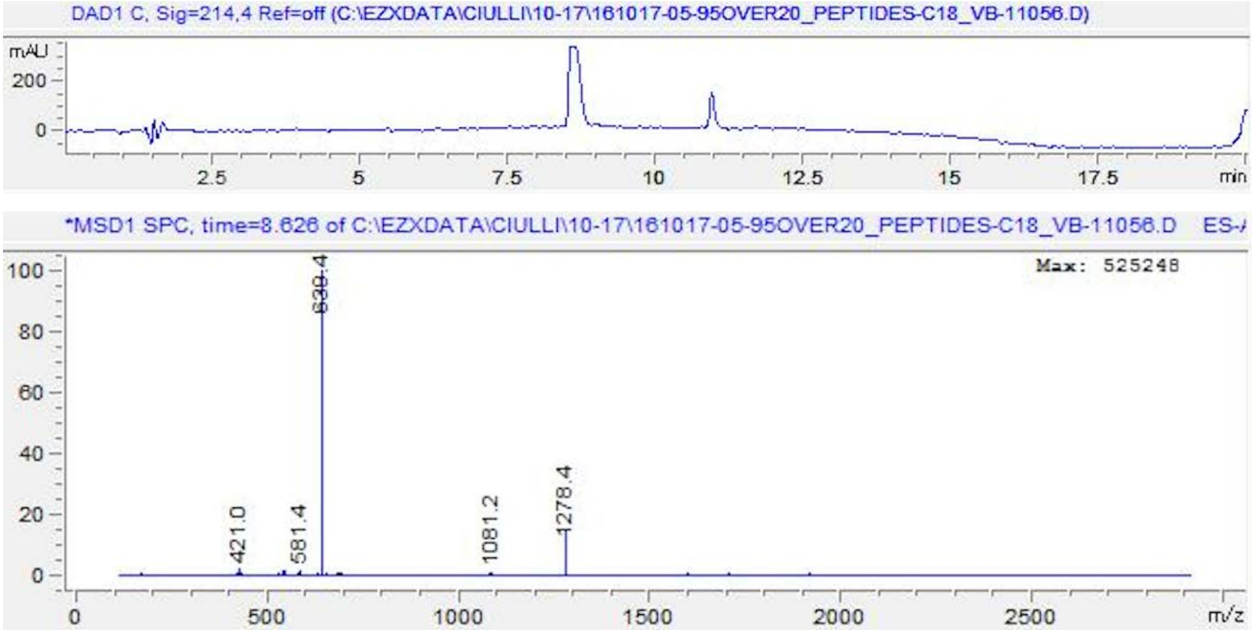

PVPDpYTSIAIV (MW~1253.575)

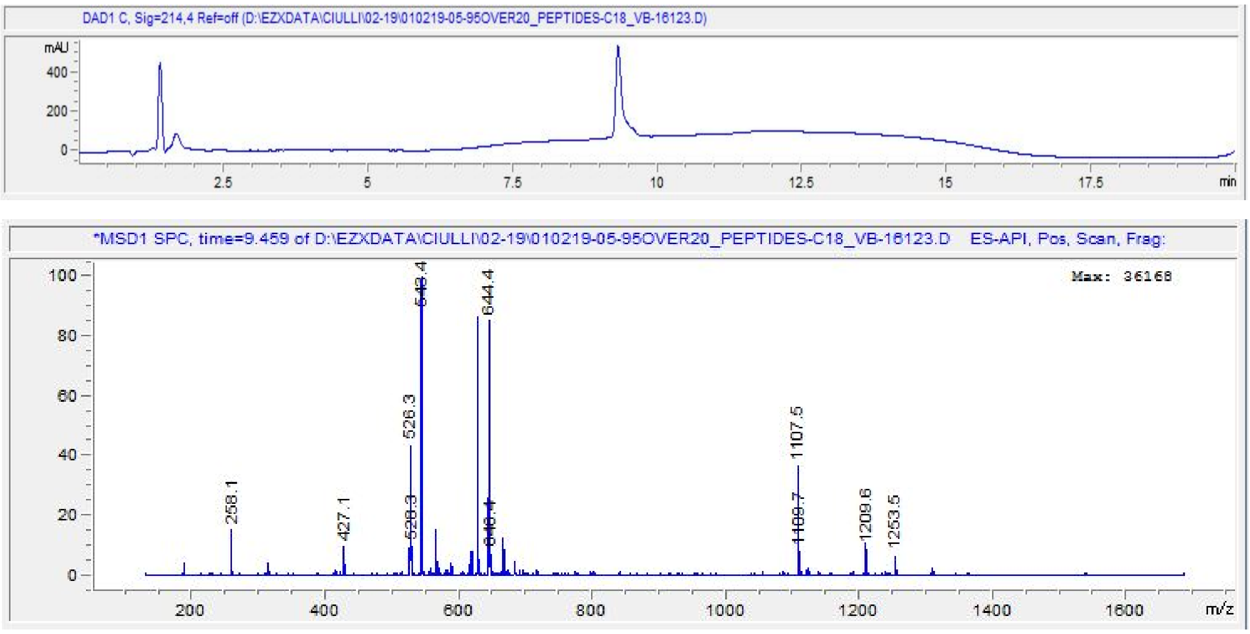

PVPDpYTSIHAV (MW~1277.550)

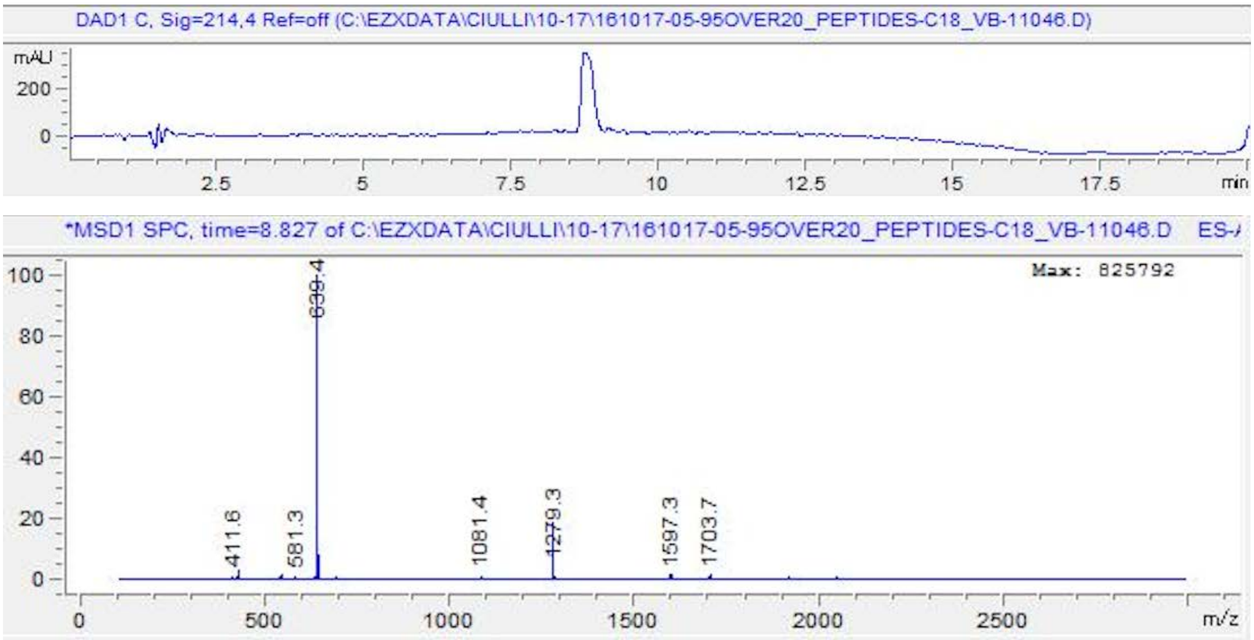

PVPDpYTSIHIA (MW~1291.565)

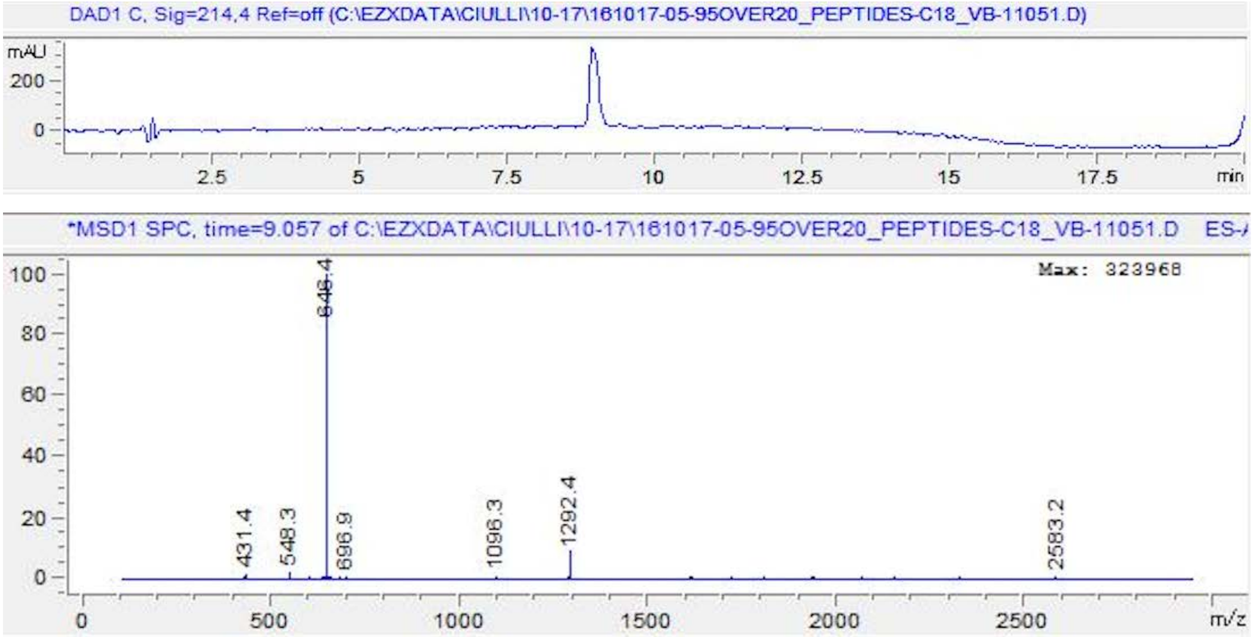

ASFEpYTILDPS (MW~1321.528)

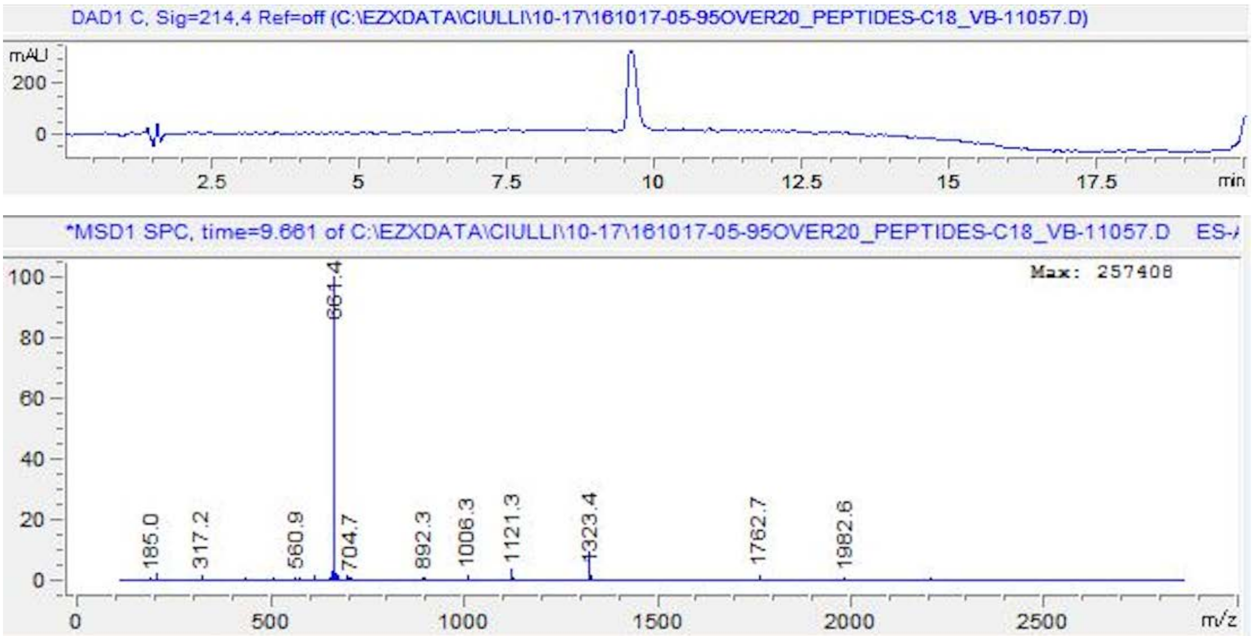

AAFEpYTILDPS (MW~1305.533)

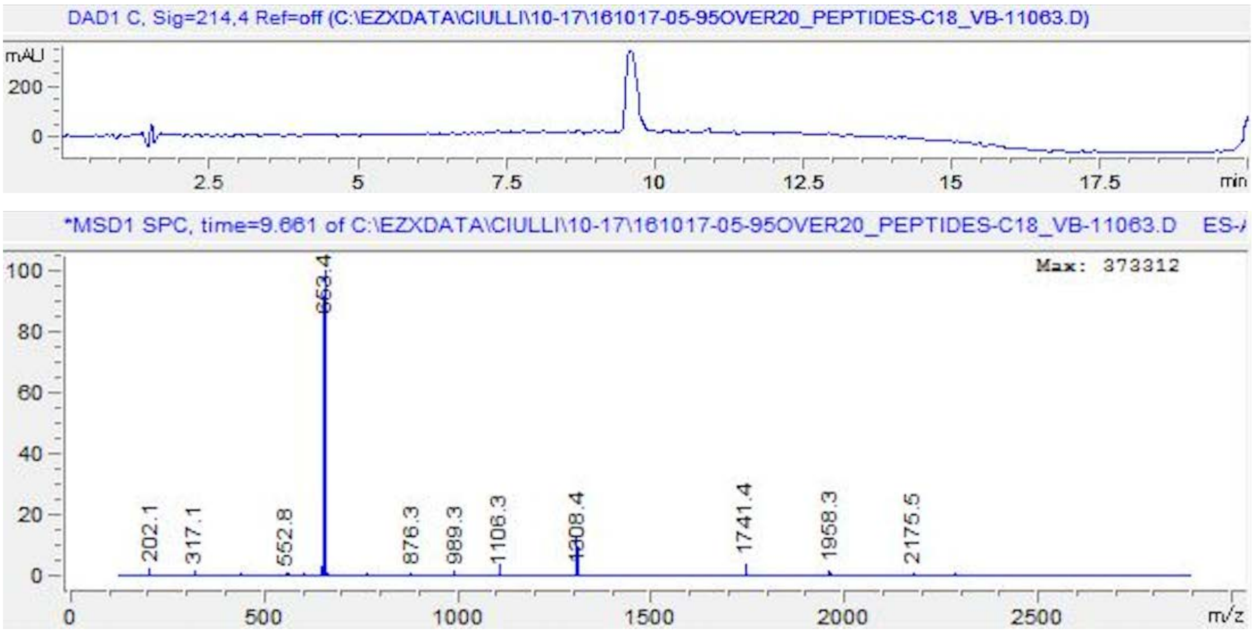

ASAEpYTILDPS (MW~1245.497)

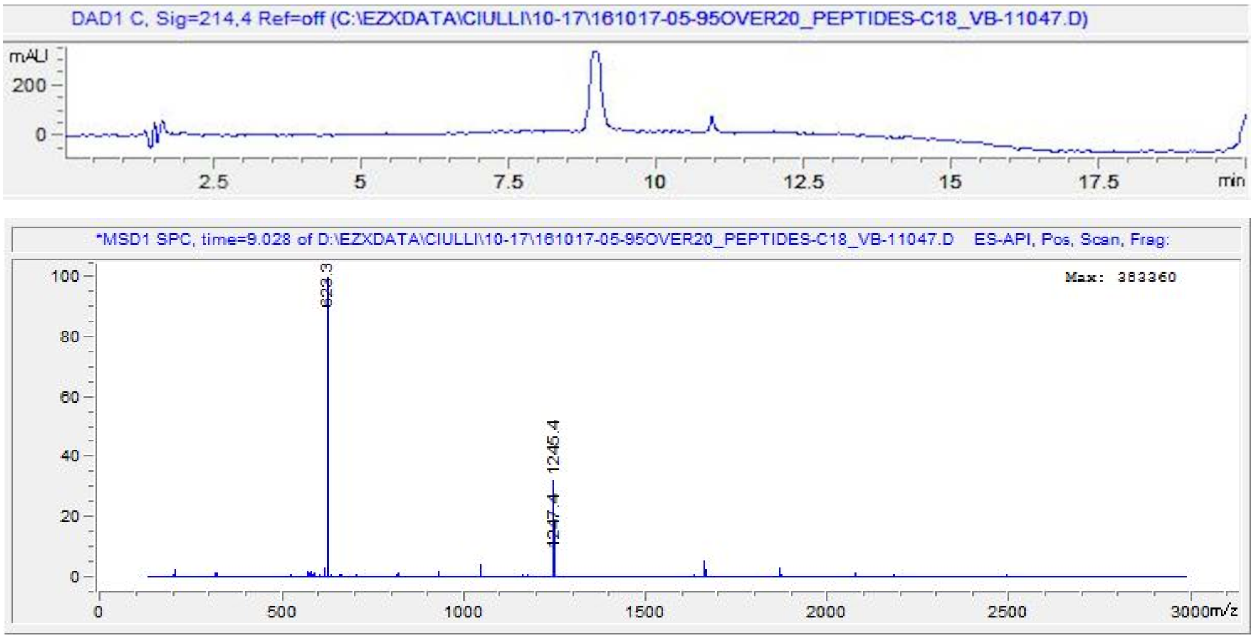

ASFApYTILDPS (MW~1263.523)

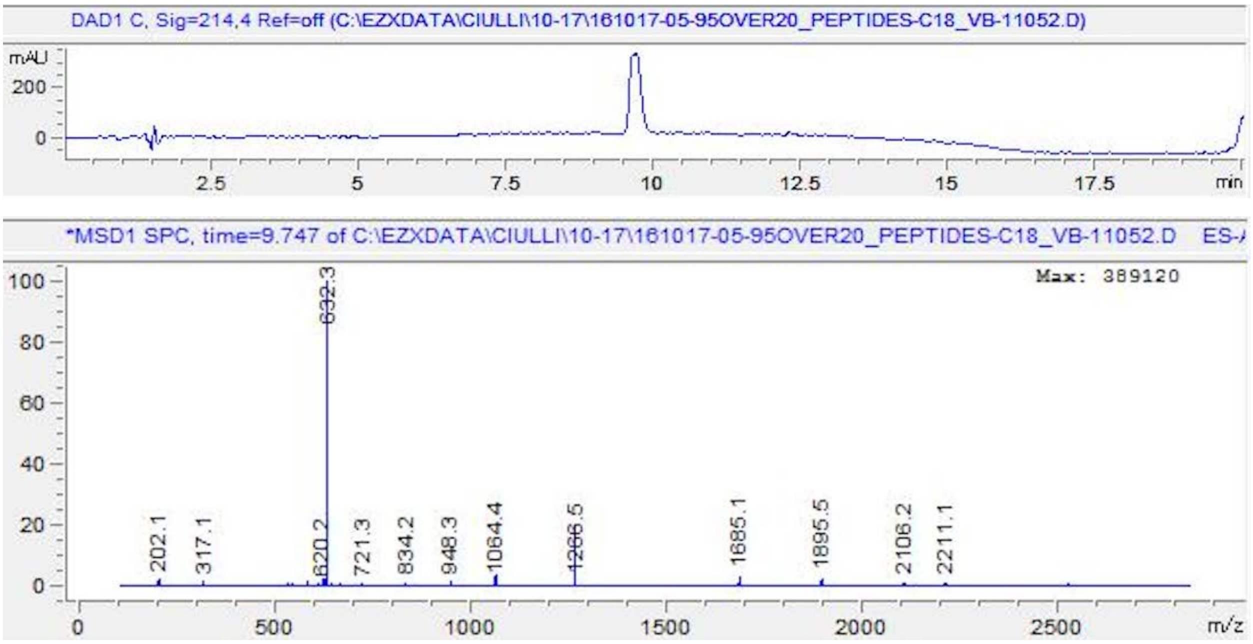

ASFEpYAILDPS (MW~1291.518)

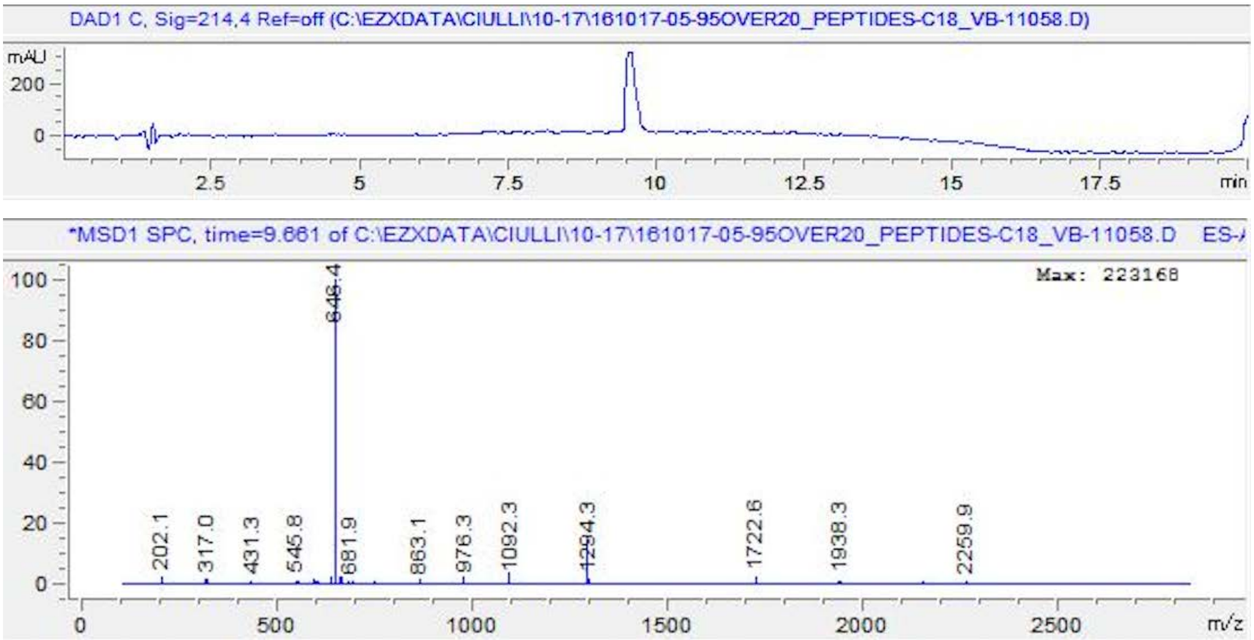

ASFEpYTALDPS (MW~1279.481)

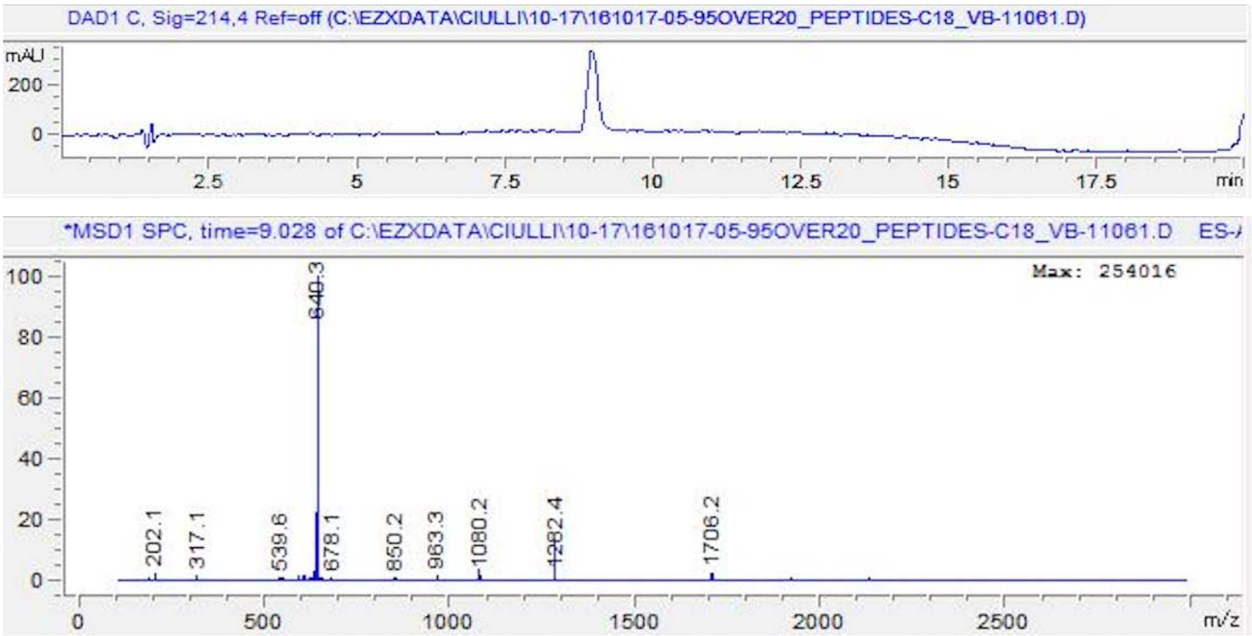

ASFEpYTIADPS (MW~1279.481)

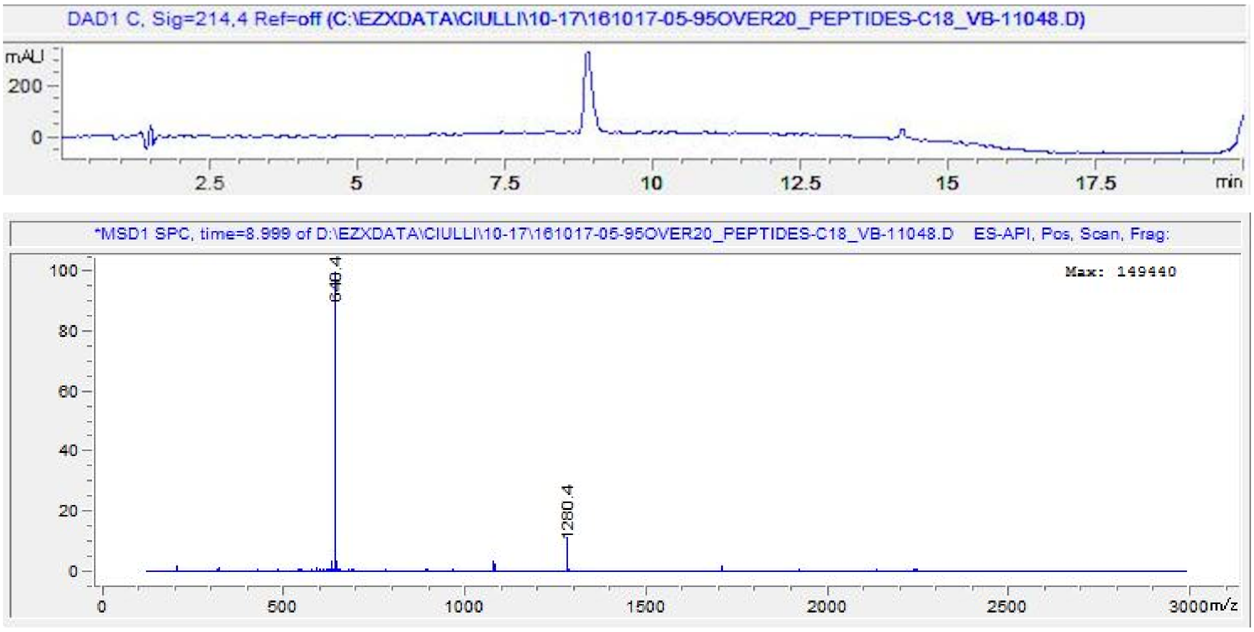

ASFEpYTILAPS (MW~1277.538)

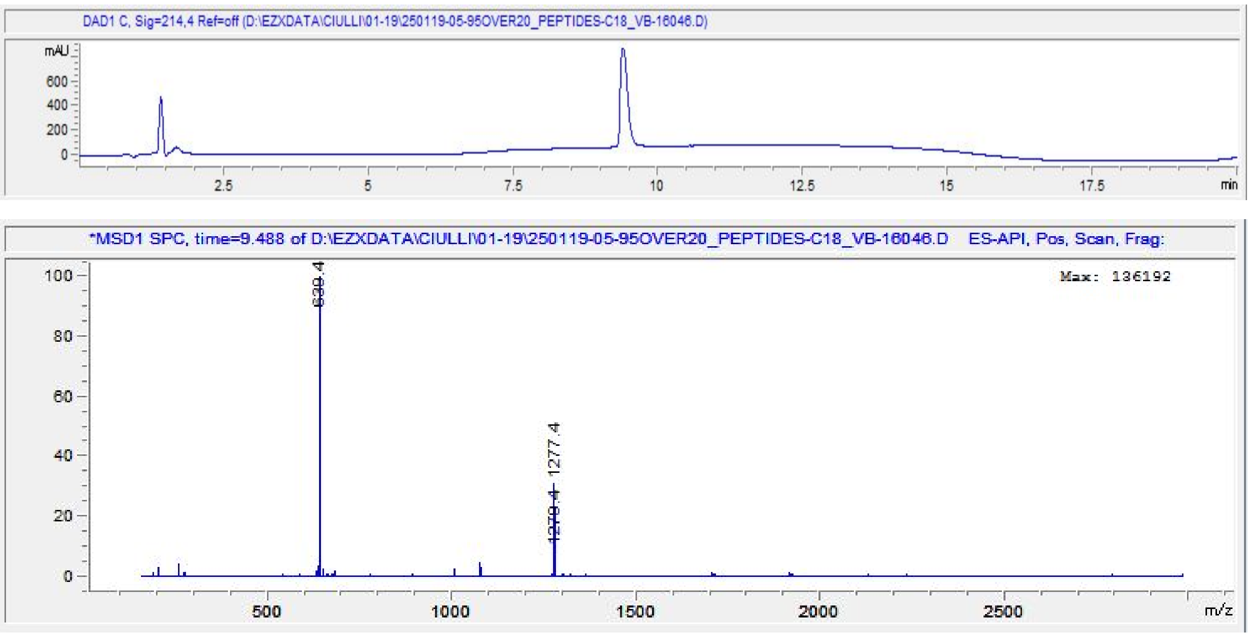

ASFEpYTILDAS (MW~1295.513)

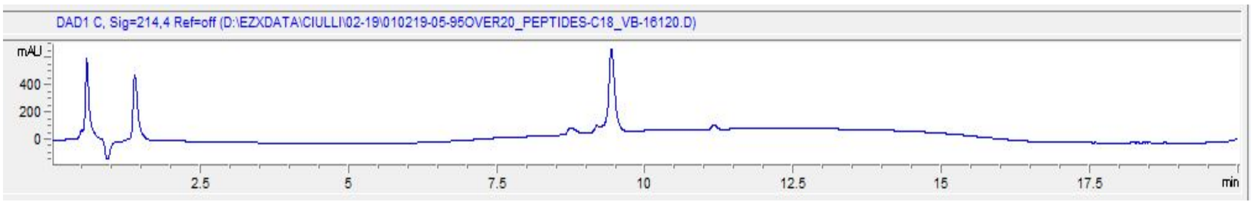

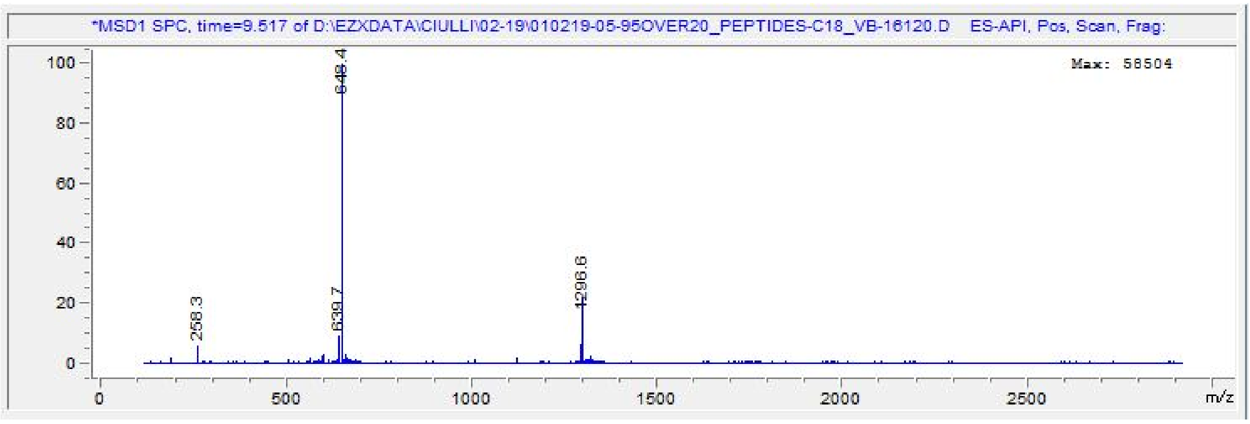

ASFEpYTILDPA (MW~1305.533)

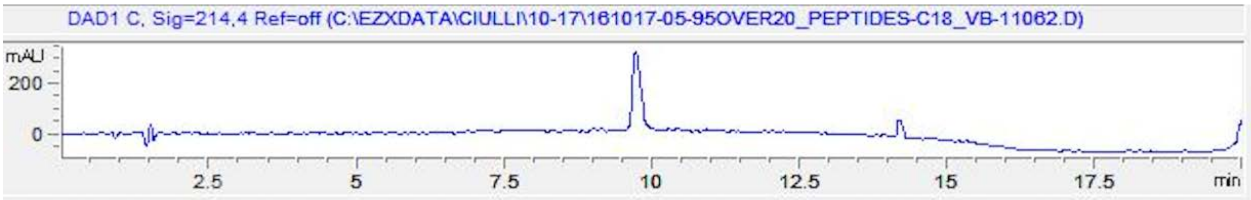

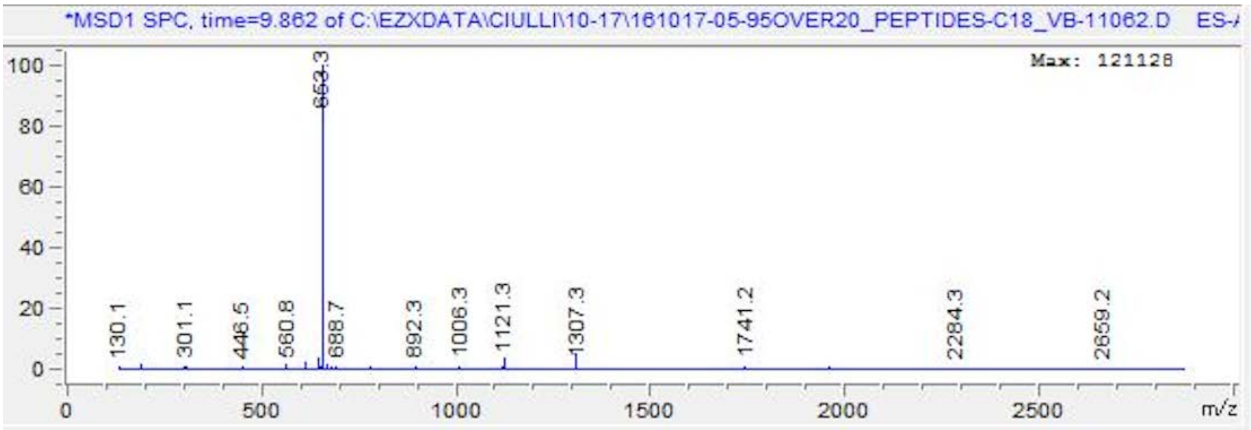

GHR mutant V(-3)R

PRPDpYTSIHIV (MW~1376.629)

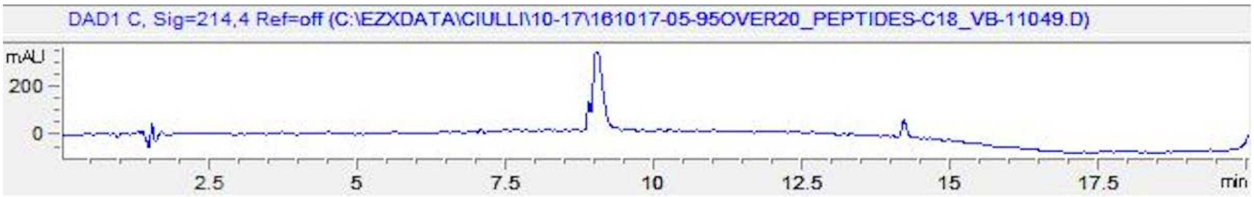

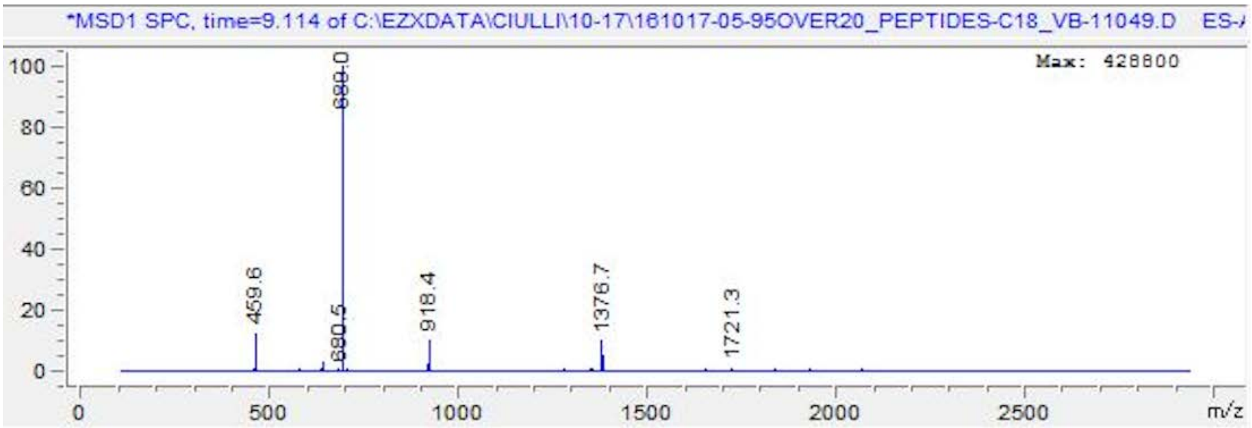

GHR mutant V(-3)Y

PYPDpYTSIHIV (MW~1383.591)

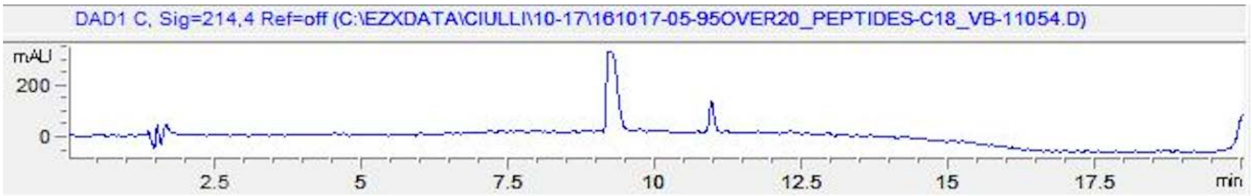

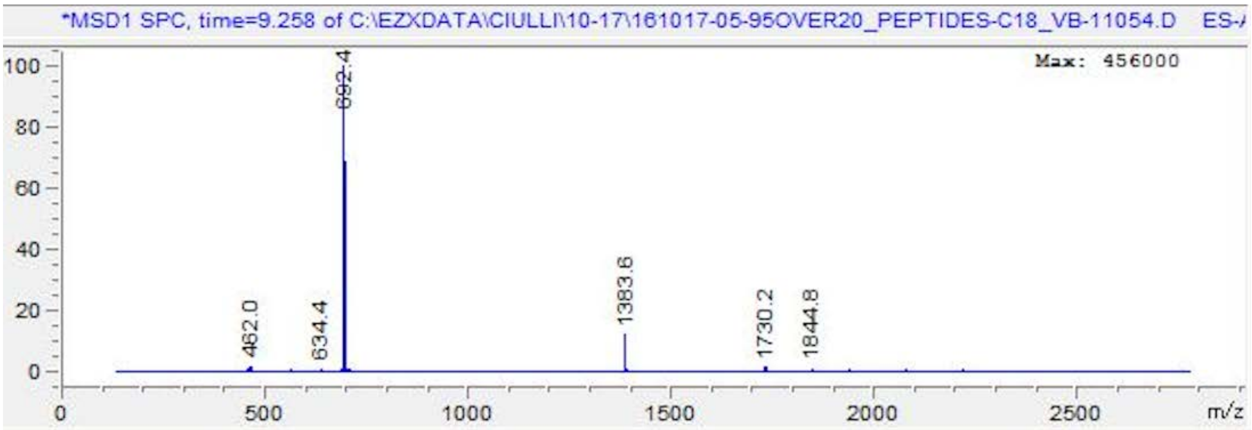

GHR_pY487 NIDFpYAQVSDI (MW~1363.550)

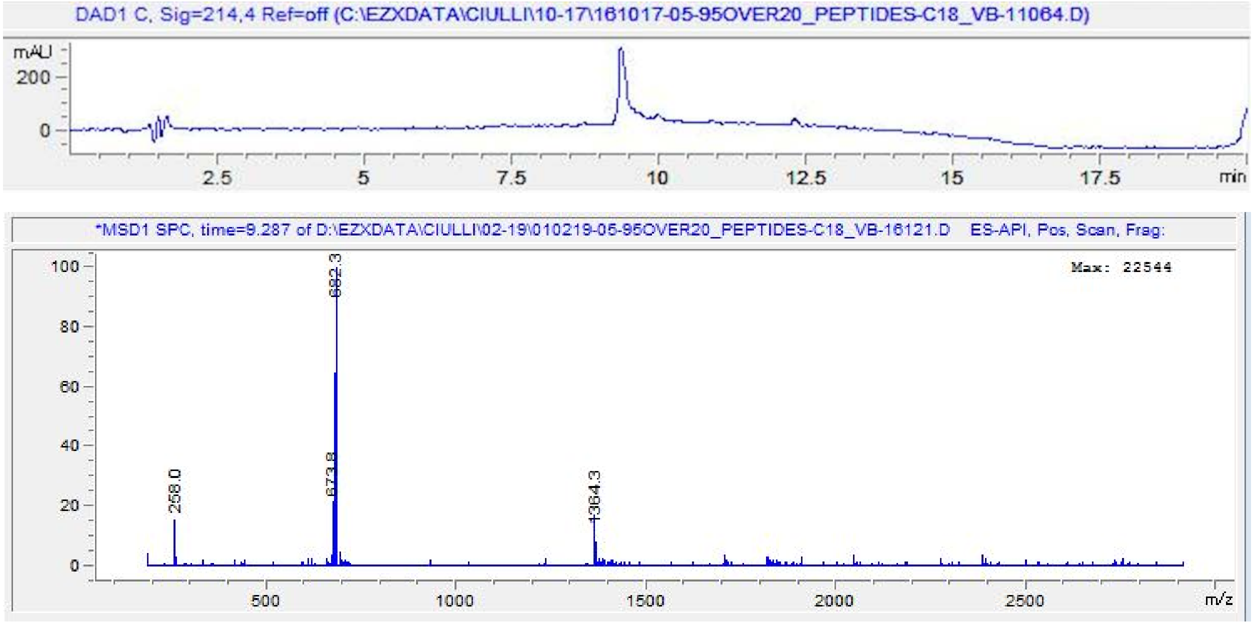

GHR_pY595 PVPDpYTSIHIV (MW~1319.597)

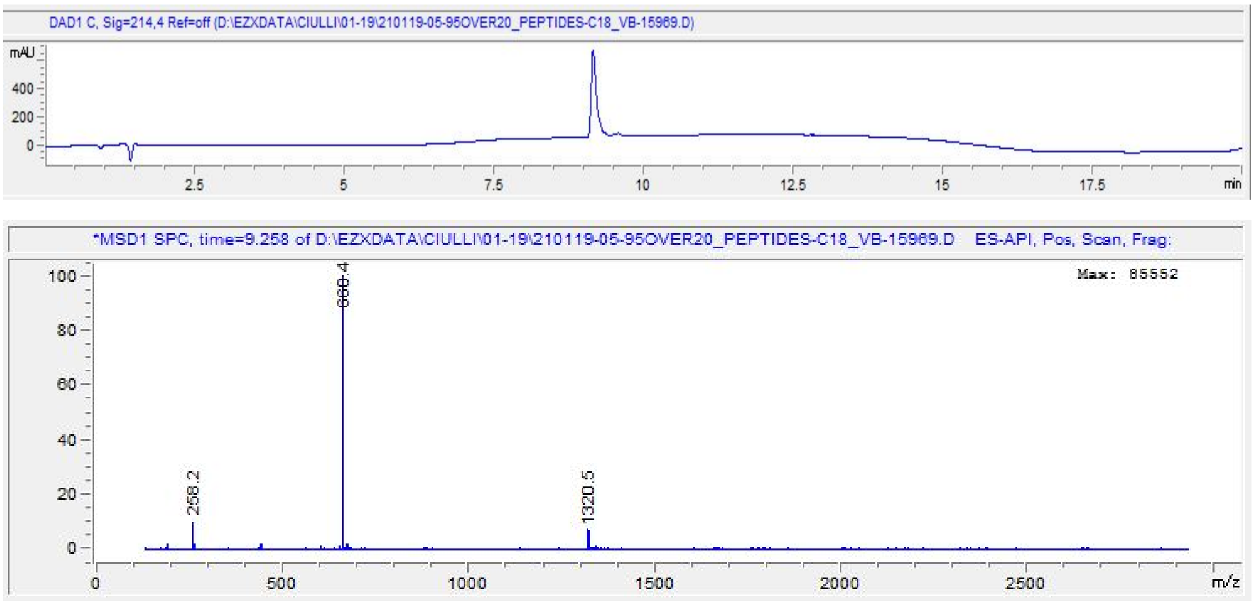

Supplementary Figure 9. The LC-MS data of synthesized peptide

Peptides were measured at concentration of 0.08, 0.25, 0.7, 2.2, 6.7 20 and 60 μM.

PVPDpYTSIHIV

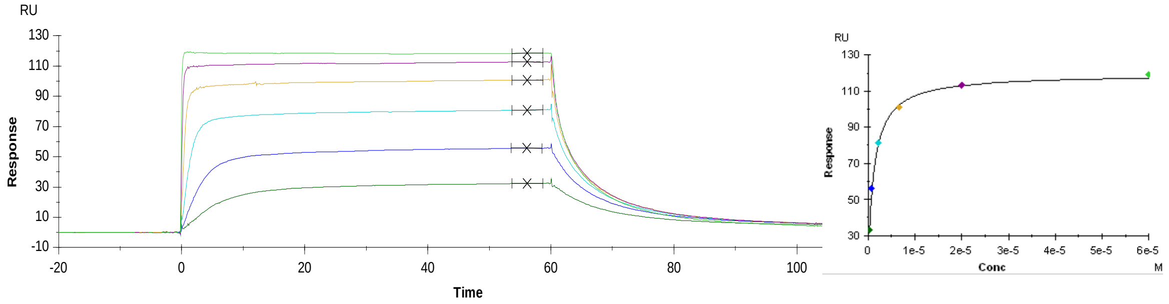

AVPDpYTSIHIV

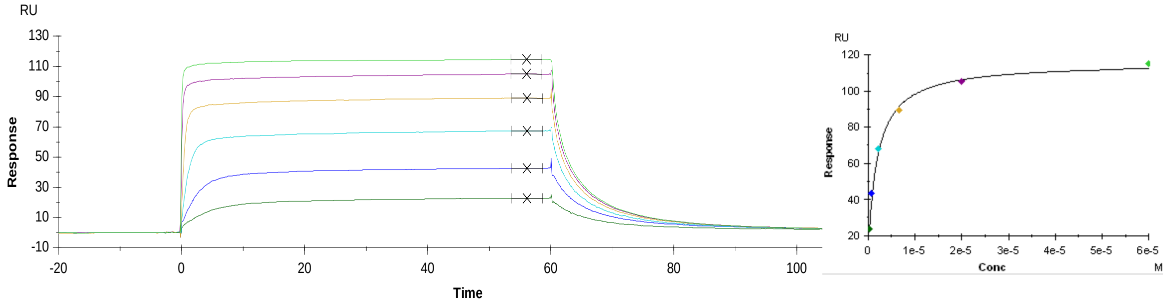

PAPDpYTSIHIV

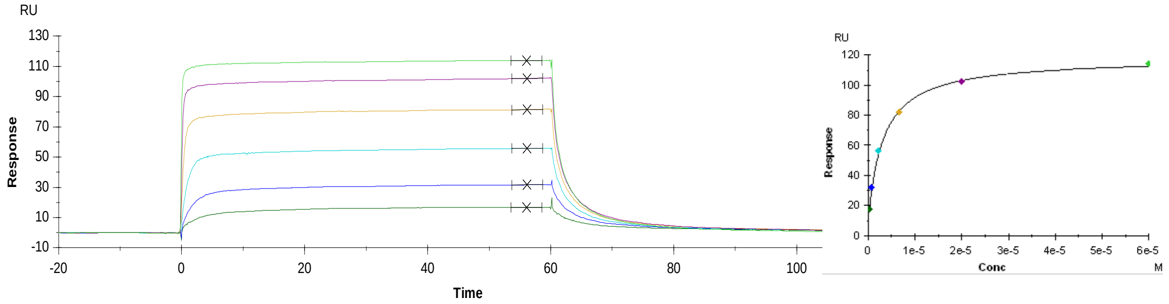

PVADpYTSIHIV

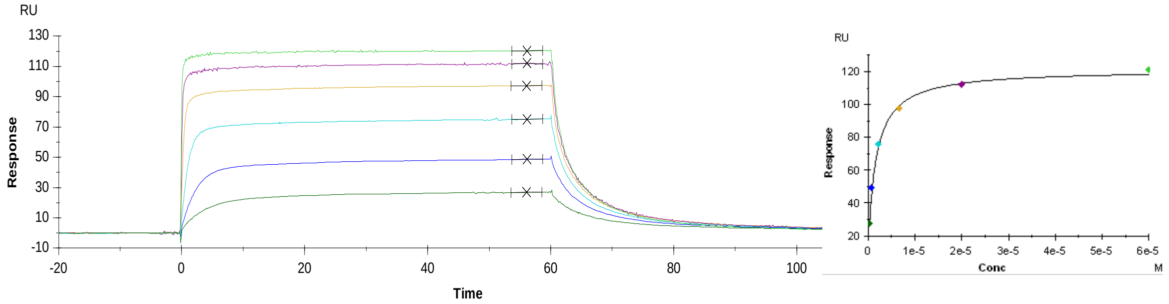

PVPApYTSIHIV

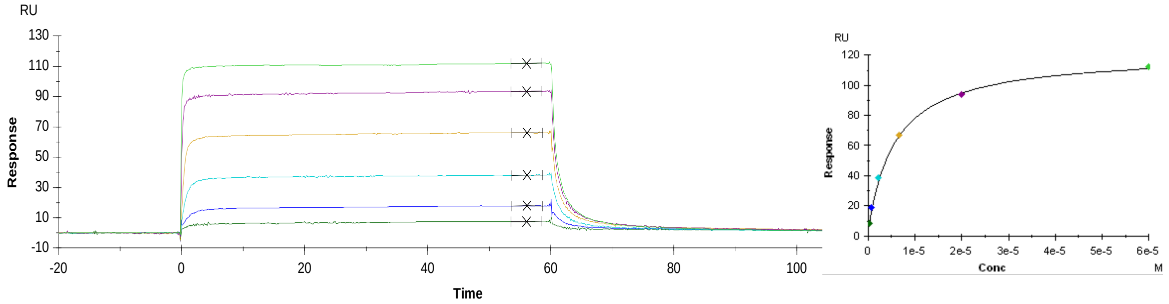

PVPDpYASIHIV

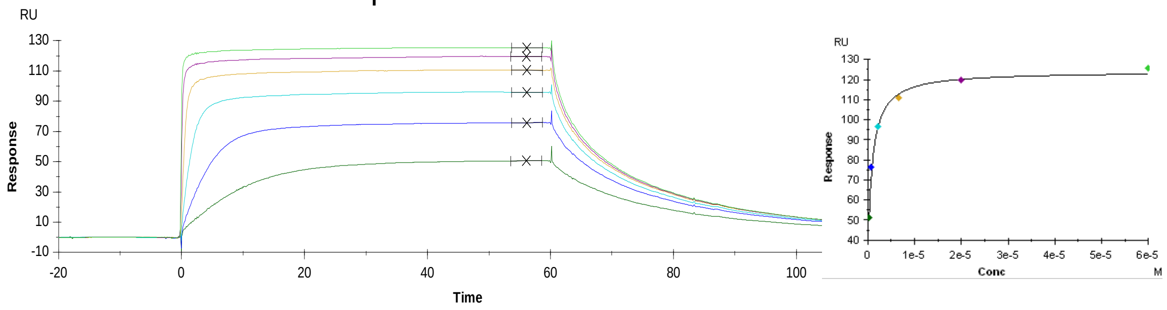

PVPDpYTAIHIV

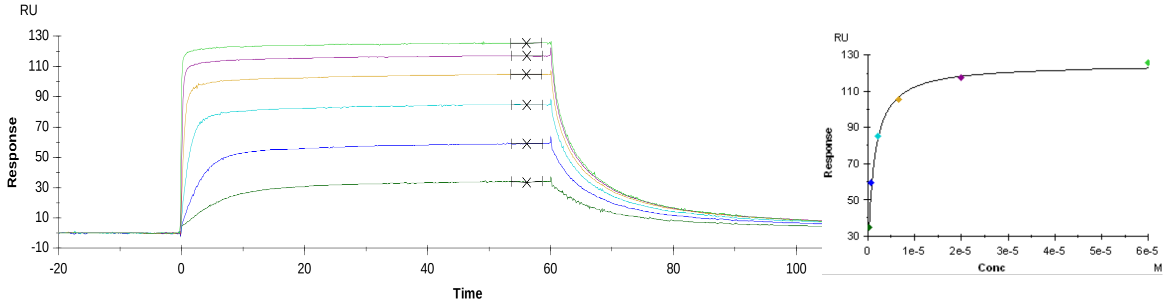

PVPDpYTSAHIV

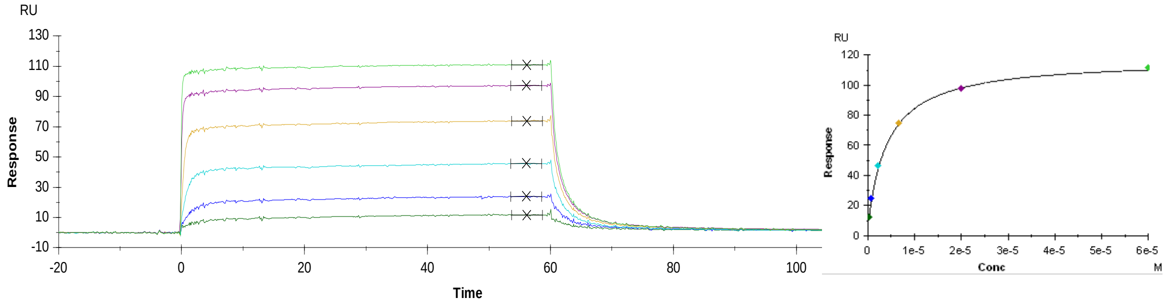

PVPDpYTSIAIV

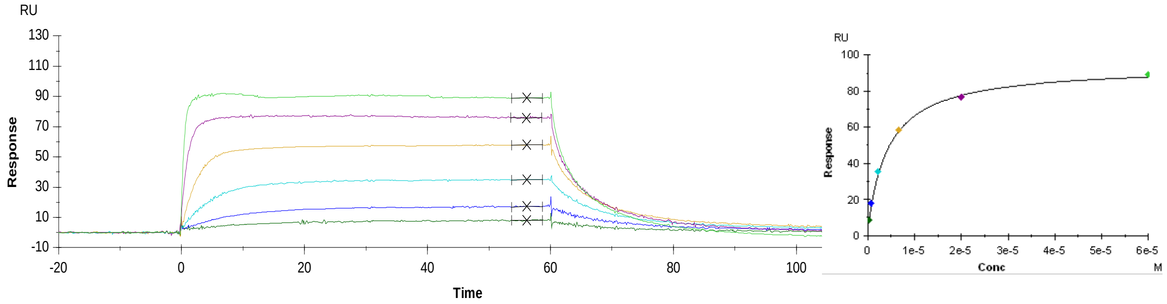

PVPDpYTSIHAV

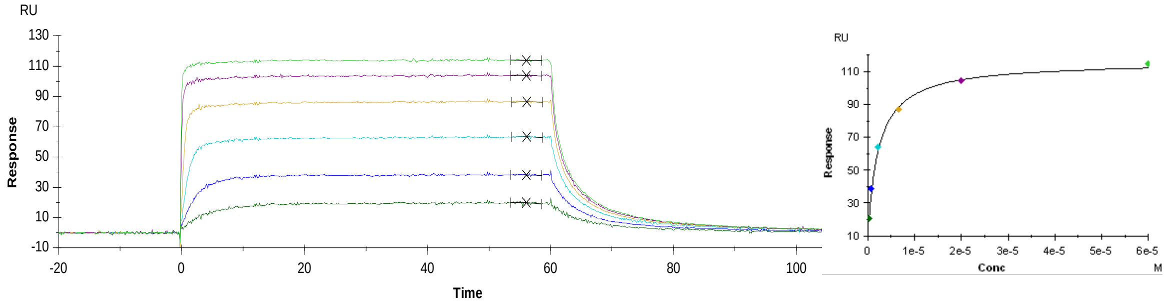

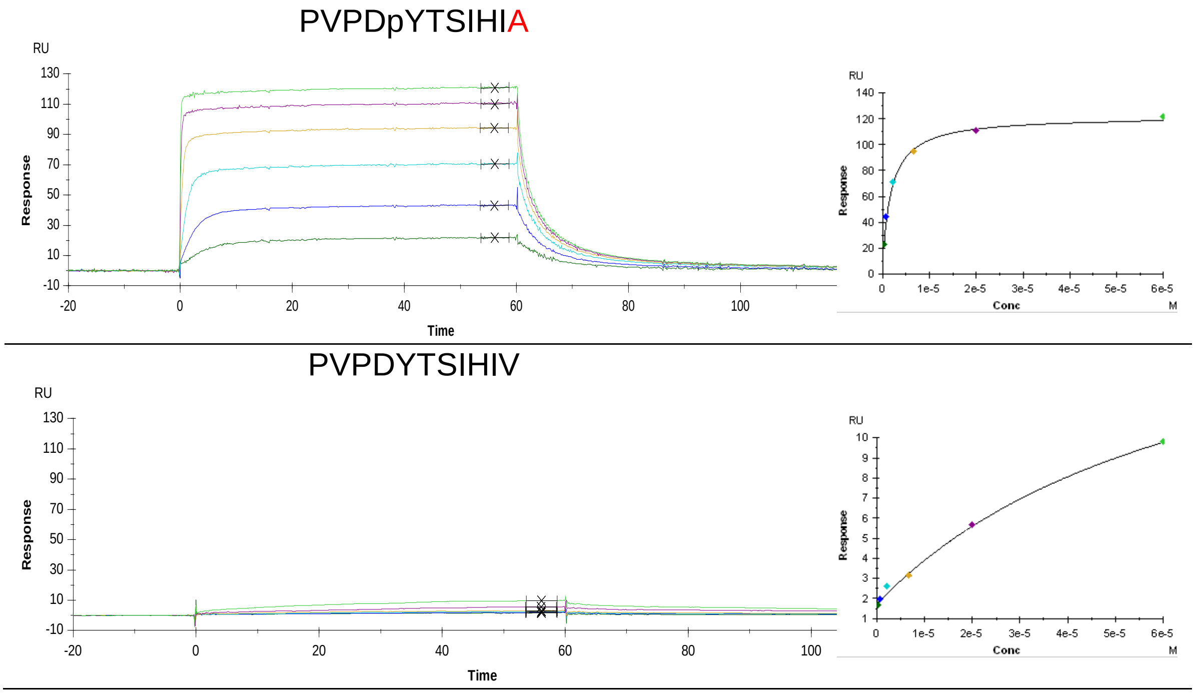

Supplementary Figure 10. Alanine scan of GHR_pY595 derivatives by SPR

Peptides were measured at concentration of 0.08, 0.25, 0.7, 2.2, 6.7 20 and 60 μM.

ASFEpYTILDPS

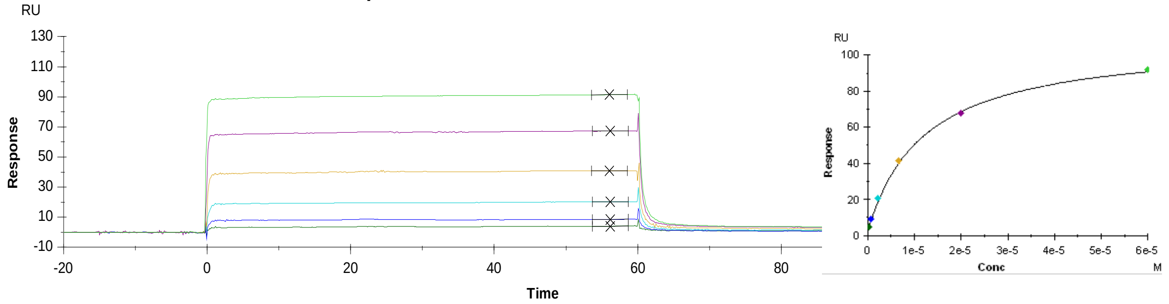

AAFEpYTILDPS

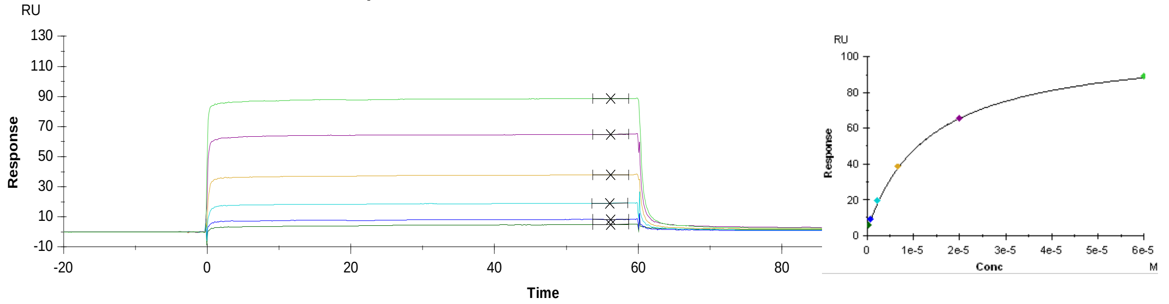

ASAEpYTILDPS

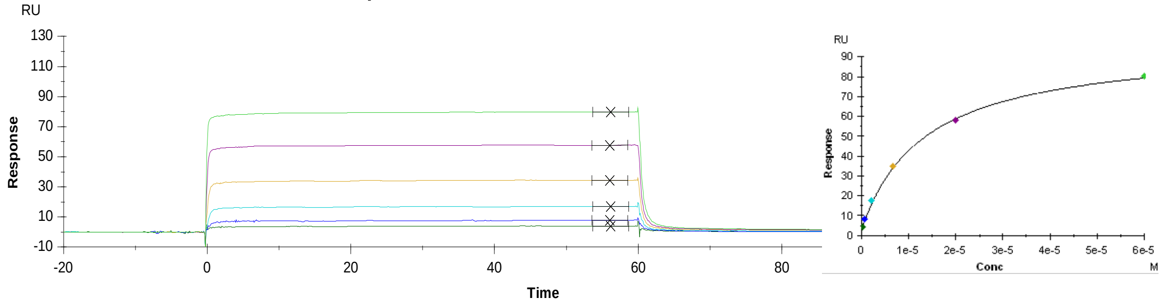

ASFApYTILDPS

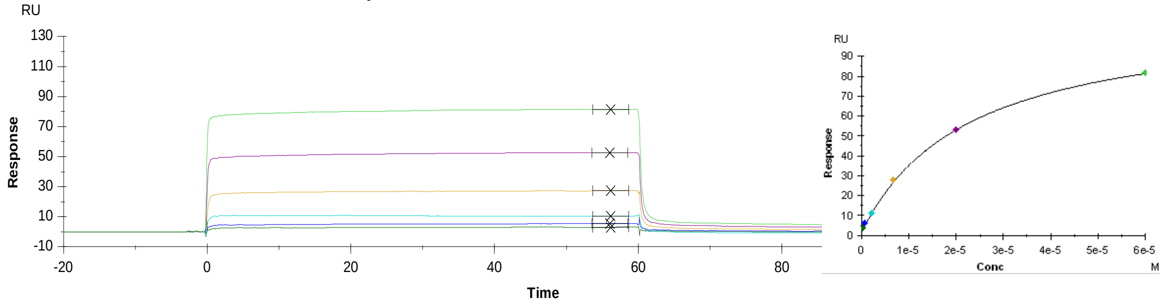

ASFEpYAILDPS

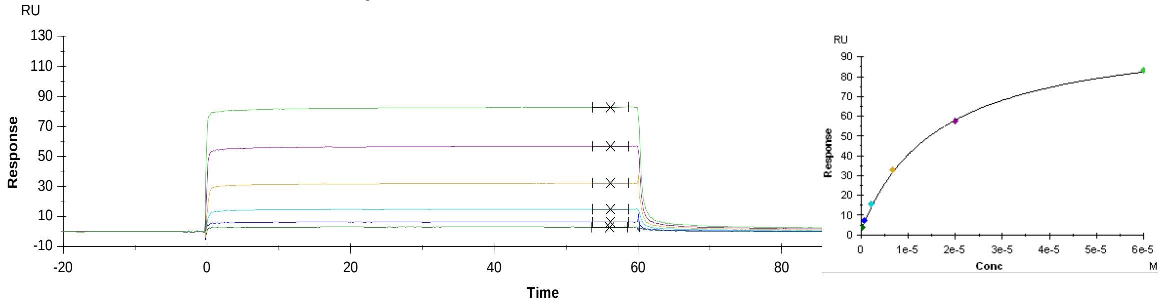

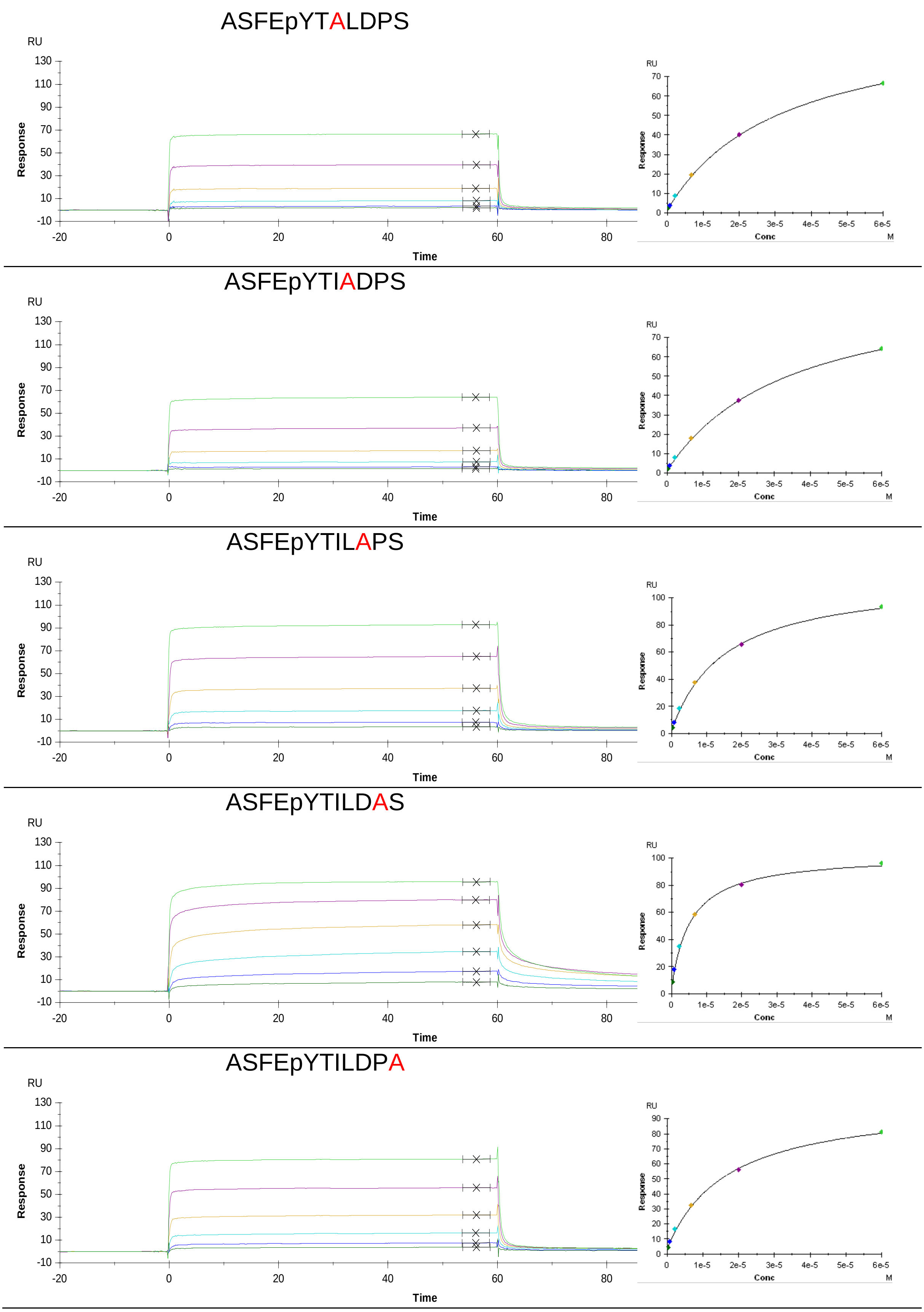

Supplementary Figure 11. Alanine scan of EpoR_pY426 derivatives by SPR

Peptides were measured at concentration of 0.08, 0.25, 0.7, 2.2, 6.7 20 and 60 μM.

PRPDpYTSIHIV

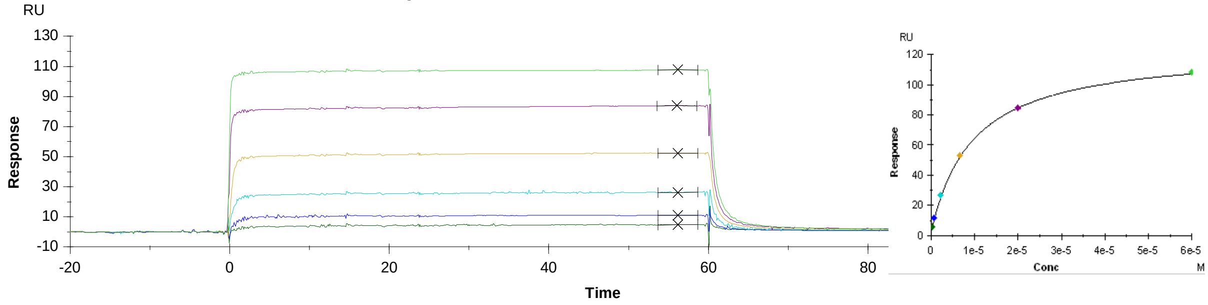

PYVPDpYTSIHIV

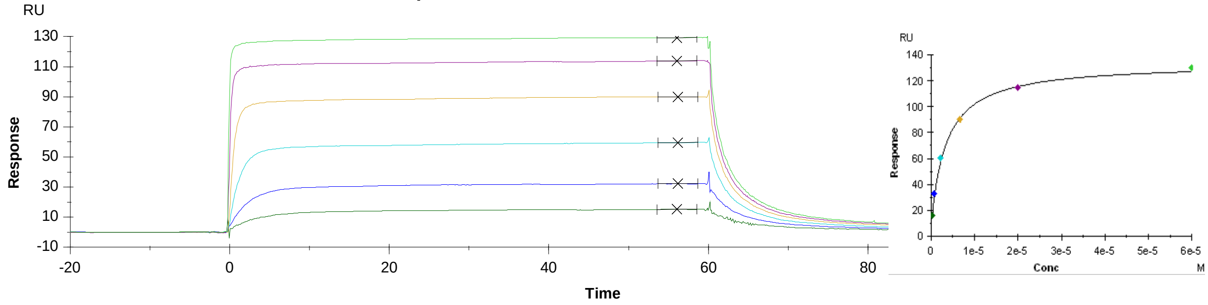

Supplementary Figure 12. K_D measurement of the GHR_pY595 with Val(-3) substituted by arginine or tyrosine

Wild type

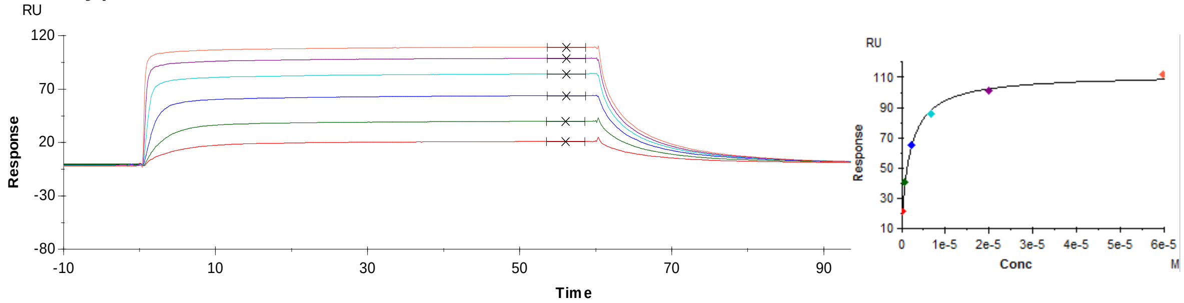

N94D

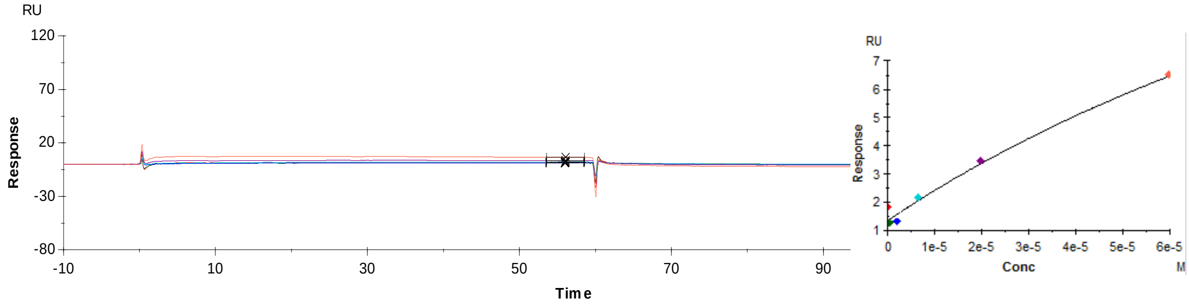

R96L

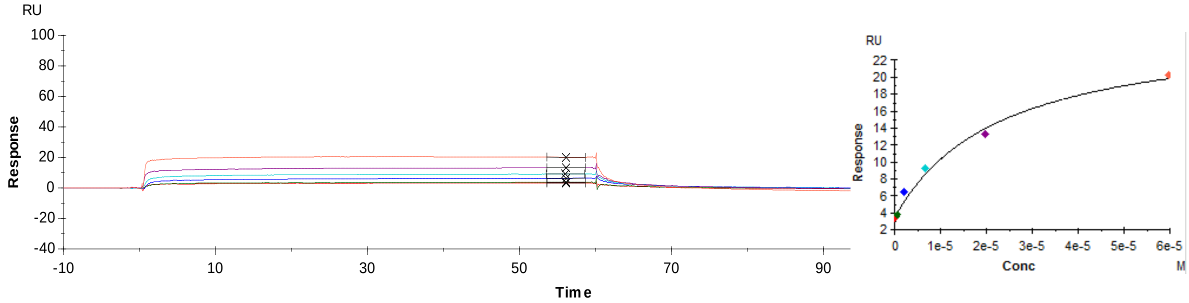

R96Q

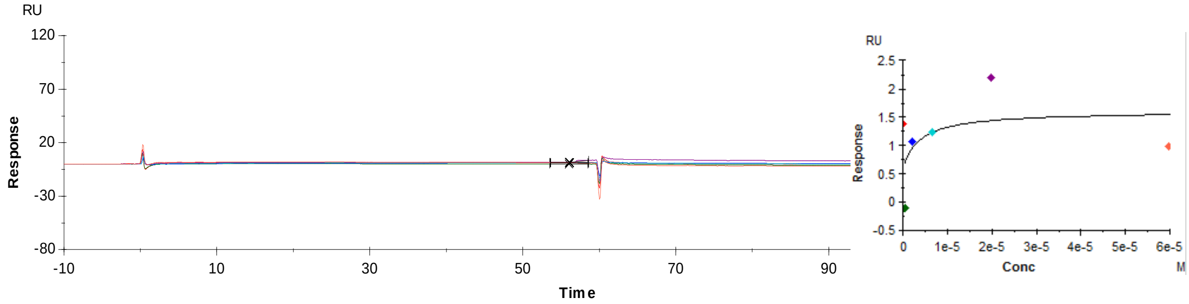

L106V

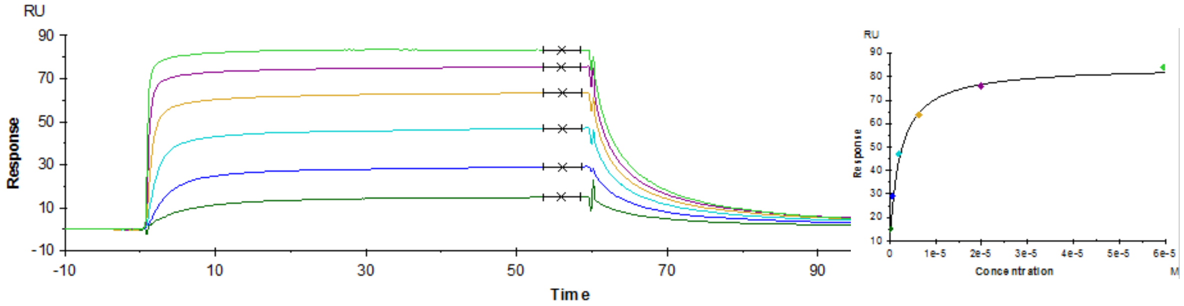

C133Y

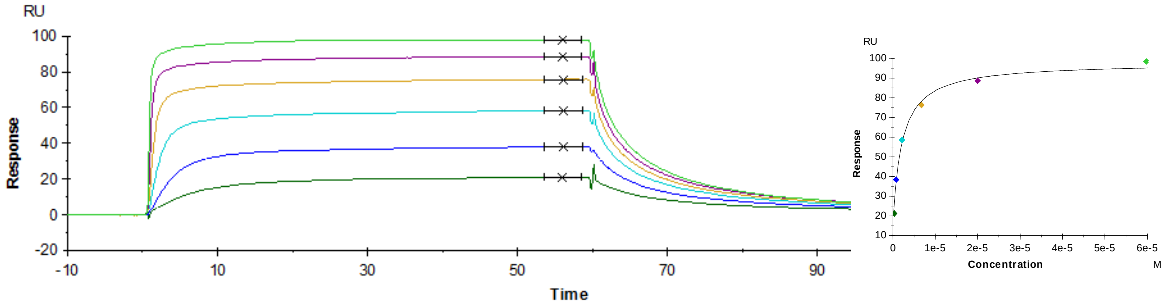

Supplementary Figure 13. The K_D measurement of GHR_pY595 peptide against SNP mutants by SPR

Peptides were measured at concentration of 0.08, 0.25, 0.7, 2.2, 6.7 20 and 60 μM.

WT

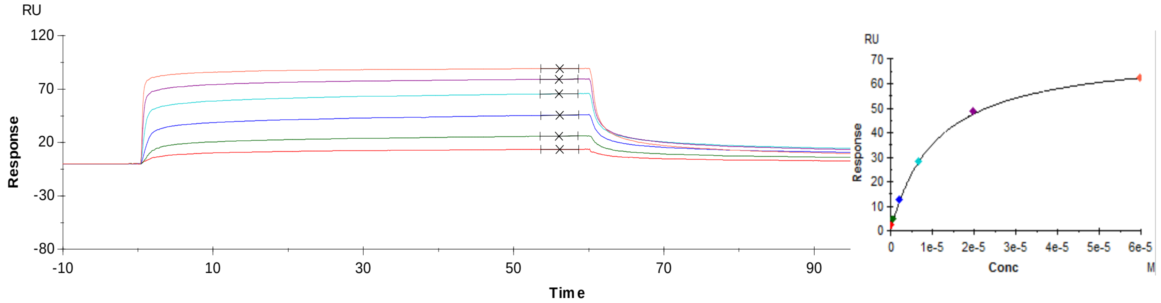

N94D

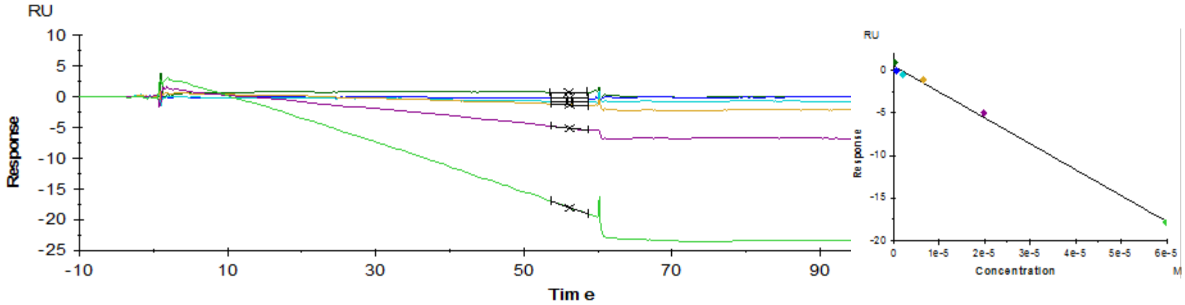

R96L

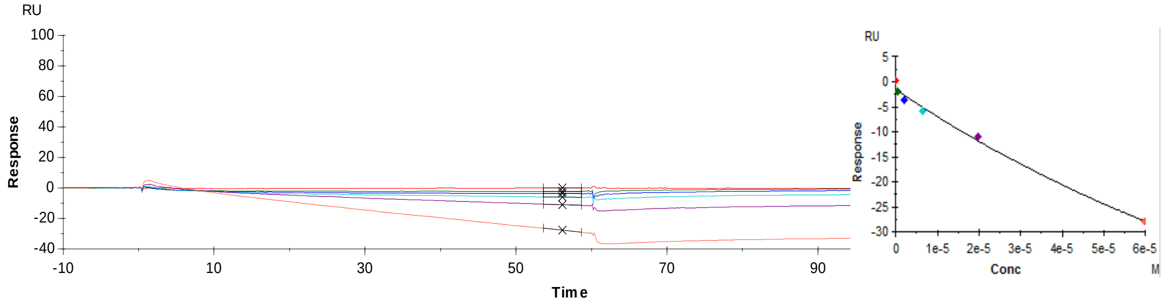

R96Q

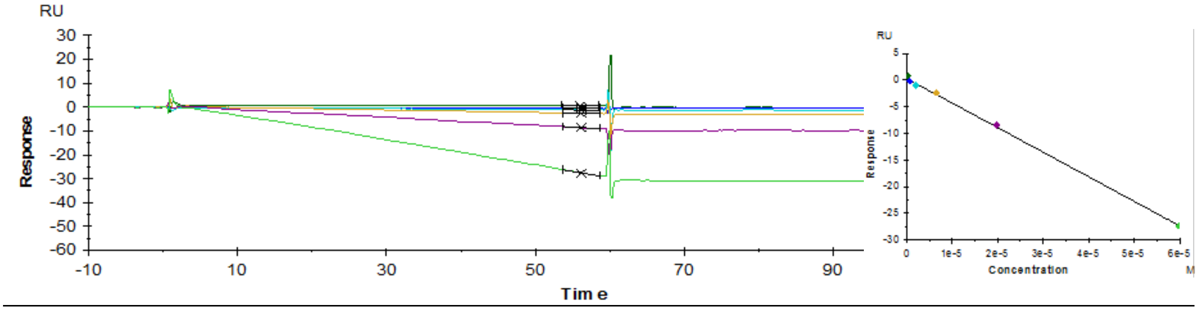

L106V

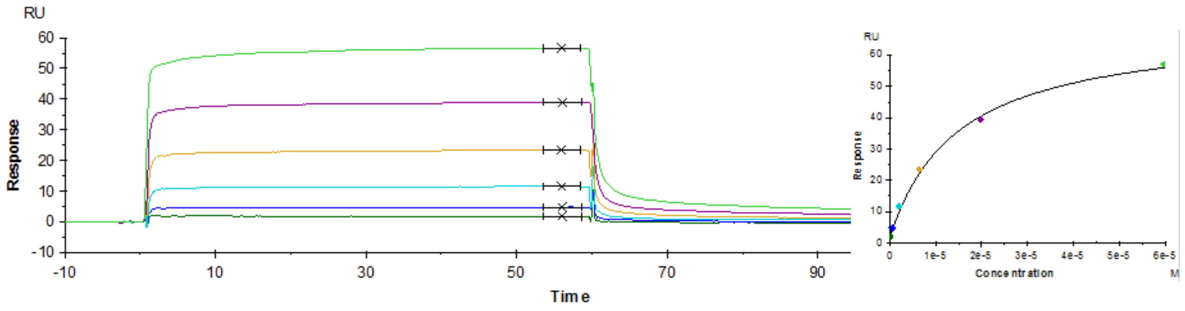

C133Y

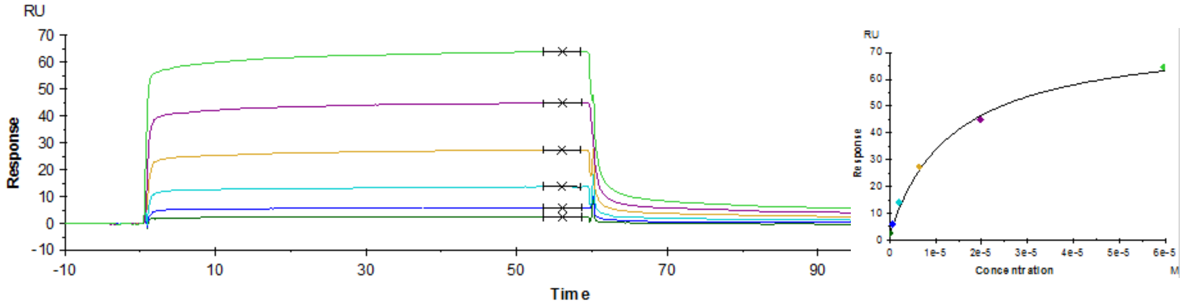

Supplementary Figure 14. The K_D measurement of EpoR_pY426 peptide against SNP mutants by SPR

Peptides were measured at concentration of 0.08, 0.25, 0.7, 2.2, 6.7 20 and 60 μM.

WT

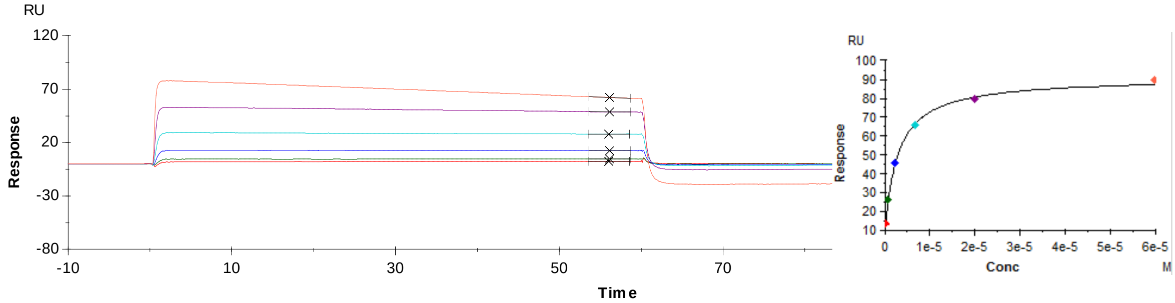

N94D

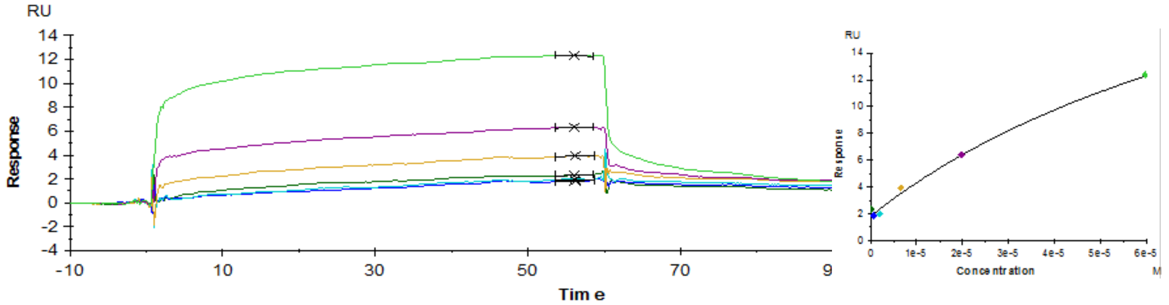

R96L

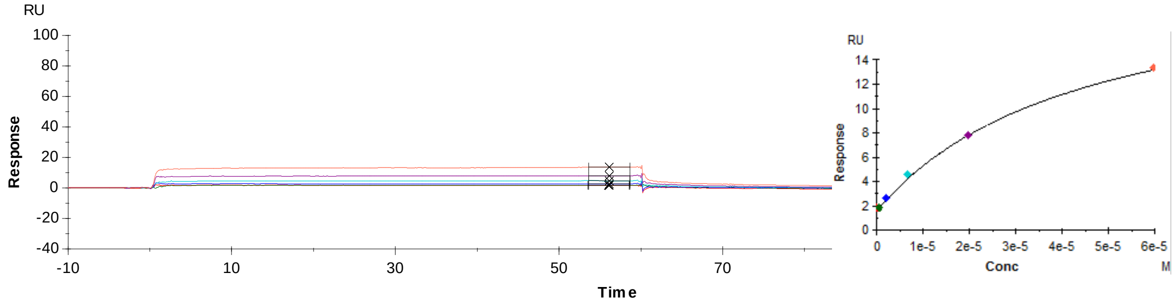

R96Q

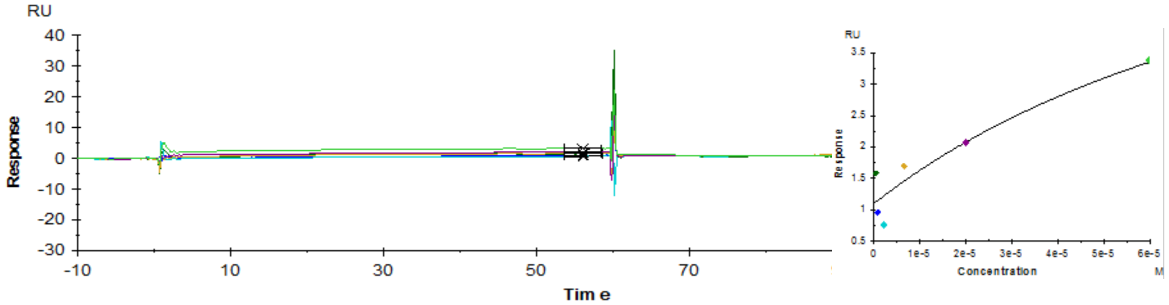

L106V

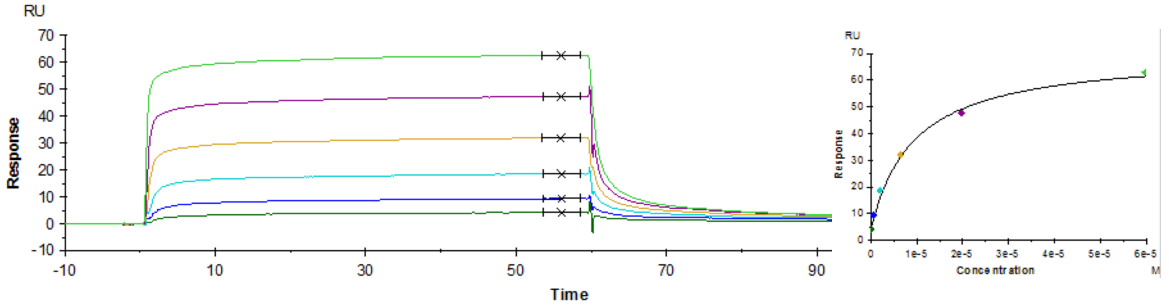

C133Y

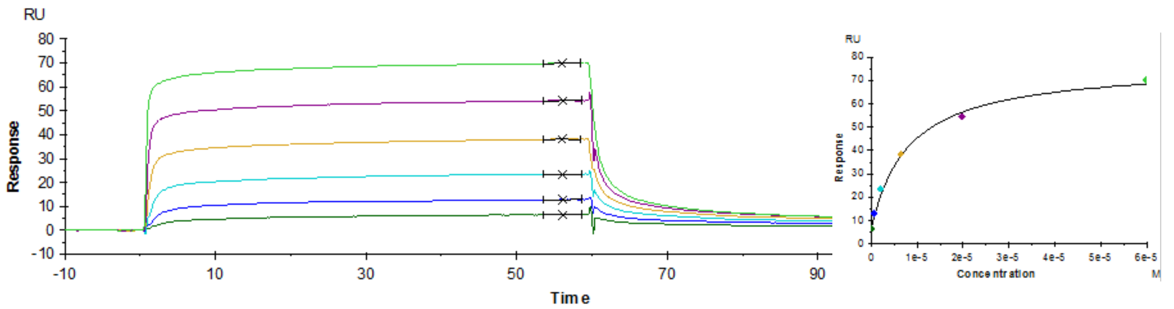

Supplementary Figure 15. The K_D measurement of GHR_pY487 peptide against SNP mutants by SPR

Peptides were measured at concentration of 0.08, 0.25, 0.7, 2.2, 6.7 20 and 60 μM.

Supplementary Table 1. Primer sequences for mutagenesis

	Forward primer (5’ -> 3’)	Reverse primer (5’ -> 3’)
SER mutation		
K115A/K117A/Q118A	ATATGTGTCAAATCCGCGCTTGCAGCATTTGACAGTGTGGTT	AACCACACTGTCAAATGCTGCAAGCGCGGATTTGACACATAT
SNPs study		
N94D	TCAGCTGGACCAACTGATCTTCGAATCGAATAC	GTATTCGATTCGAAGATCAGTTGGTCCAGCTGA
R96Q	GGACCAACTAATCTTCAAATCGAATACCAAGAC	GTCTTGGTATTCGATTTGAAGATTAGTTGGTCC
R96L	GGACCAACTAATCTTCTAATCGAATACCAAGAC	GTCTTGGTATTCGATTAGAAGATTAGTTGGTCC
L106V	GACGGAAAAATTCAGAGTGGACTCTATCATATGT	ACATATGATAGAGTCCACTCTGAATTTTCCGTC
C133Y	TACTATGTTCAGATGTACAAGGATAAGCGGACA	TGTCCGCTTATCCTTGTACATCTGAACATAGTA
